# Diagnostic accuracy of two multiplex real-time polymerase chain reaction assays for the diagnosis of meningitis in children in a resource-limited setting

**DOI:** 10.1371/journal.pone.0173948

**Published:** 2017-03-27

**Authors:** Jermaine Khumalo, Mark Nicol, Diana Hardie, Rudzani Muloiwa, Phindile Mteshana, Colleen Bamford

**Affiliations:** 1 Division of Medical Microbiology, Department of Pathology, University of Cape Town, Cape Town, South Africa; 2 National Health Laboratory Service, Johannesburg, South Africa; 3 Division of Virology, Department of Pathology, University of Cape Town, Cape Town, South Africa; 4 Department of Paediatrics, University of Cape Town, Cape Town, South Africa; Wadsworth Center, UNITED STATES

## Abstract

**Introduction:**

Accurate etiological diagnosis of meningitis is important, but difficult in resource-limited settings due to prior administration of antibiotics and lack of viral diagnostics. We aimed to develop and validate 2 real-time multiplex PCR (RT-PCR) assays for the detection of common causes of community-acquired bacterial and viral meningitis in South African children.

**Methods:**

We developed 2 multiplex RT- PCRs for detection of *S*. *pneumoniae*, *N*. *meningitidis*, *H*. *influenzae*, enteroviruses, mumps virus and herpes simplex virus. We tested residual CSF samples from children presenting to a local paediatric hospital over a one-year period, whose CSF showed an abnormal cell count. Results were compared with routine diagnostic tests and the final discharge diagnosis. We calculated accuracy of the bacterial RT-PCR assay compared to CSF culture and using World Health Organisation definitions of laboratory-confirmed bacterial meningitis.

**Results:**

From 292 samples, bacterial DNA was detected in 12 (4.1%) and viral nucleic acids in 94 (32%). Compared to CSF culture, the sensitivity and specificity of the bacterial RT-PCR was 100% and 97.2% with complete agreement in organism identification. None of the cases positive by viral RT-PCR had a bacterial cause confirmed on CSF culture. Only 9/90 (10%) of patients diagnosed clinically as bacterial meningitis or partially treated bacterial meningitis tested positive with the bacterial RT-PCR.

**Discussion:**

In this population the use of 2 multiplex RT-PCRs targeting 6 common pathogens gave promising results. If introduced into routine diagnostic testing, these multiplex RT-PCR assays would supplement other diagnostic tests, and have the potential to limit unnecessary antibiotic therapy and hospitalisation.

## Introduction

Bacterial meningitis (BM) is a life threatening illness in children[1]. A high index of suspicion and rapid initiation of appropriate antibiotics are necessary to minimise adverse outcomes, and various child health programmes now recommend pre-hospital administration of broad spectrum antibiotics in cases of suspected sepsis or meningitis[2, 3]. The diagnosis of bacterial meningitis is traditionally confirmed by microbiological testing of cerebro-spinal fluid (CSF), which includes cytochemical analysis, cell count, microscopy and culture[4]. However, administration of antibiotics prior to lumbar puncture may decrease the yield of culture, creating diagnostic uncertainty[5]. In many routine settings, the lack of availability or lack of use of diagnostic tests for viral causes of meningitis compounds the problem[4, 6, 7]. Inability to distinguish between partially treated bacterial meningitis and viral meningitis can lead to unnecessarily prolonged antibiotic treatment and increased number and duration of hospital admissions [8, 9].

Molecular diagnostic tests, including polymerase chain reaction (PCR), can improve the diagnosis of infectious diseases by rapid detection of microbial nucleic acids, including from non-viable organisms[10]. PCR may be more sensitive than culture for the diagnosis of meningitis, especially where prior antibiotic treatment reduces the sensitivity of culture [6, 11]. There are a number of advantages to using real-time PCR (RT-PCR) methods as compared to conventional endpoint PCR methods, namely elimination of the need for post-amplification processing, minimisation of laboratory contamination, and a more rapid turn-around time. In addition, the development of multiplex RT-PCR assays provides the opportunity to detect multiple potential pathogens simultaneously[11–18]. Such multiplex RT-PCR assays can offer a very comprehensive panel of potential pathogens allowing for an extensive and exhaustive investigation of patients with suspected meningitis[19, 20]. However, in practice in specific geographic settings and in particular patient population groups, a far more limited number of pathogens constitute the vast majority of cases of meningitis. The inclusion of a large number of target pathogens into commercial or in-house multiplex-PCR assays increases the cost and complexity of such assays. [19, 20] and may prevent implementation of these in routine laboratory testing in low to middle income countries.

In the light of these concerns and limitations, we aimed to develop and validate 2 RT-PCR assays suitable for the detection of the commonest causes of community acquired bacterial and viral meningitis in children in South Africa. [21] [22, 23] [24–26]. Such RT-PCRs could be included in routine laboratory testing algorithms and would aid clinicians in the accurate diagnosis of meningitis. The organisms included in the bacterial RT-PCR were *S*. *pneumoniae*, *N*. *meningitidis* and *H*. *influenzae*, and in the viral RT-PCR enteroviruses, mumps and herpes simplex virus. Following initial laboratory validation we conducted a retrospective laboratory based comparison of the RT-PCR assays with the diagnostic tests routinely used in our laboratory.

## Methods

The study complies with updated STARD (Standards for Reporting Diagnostic Accuracy) guidelines intended to improve the transparency and completeness of the reporting of diagnostic accuracy studies.[27].

### Development, optimisation and validation of the multiplex assays

We adopted a previously published real-time multiplex PCR for detection of *S*. *pneumoniae*, *N*. *meningitidis* and *H*. *influenzae* targeting the *lytA*, *ctrA* and *hpd* genes respectively [28] and developed a real-time multiplex PCR for the detection of enteroviruses, mumps virus and herpes simplex virus, based on previously described real-time singleplex assays that detected the 5’UTR region[8, 29], fusion protein[30] and UL30[31] genes respectively (S1 Table).

For the bacterial multiplex, we used SensiFAST^™^ Probe No-ROX kit (Bioline, London, United Kingdom) and 2μl of DNA template, and for the viral multiplex iScript One Step RT-PCR Kit (Bio-Rad Laboratories Inc., Hercules, CA, United States of America) with 2μl of extracted nucleic acid. The concentrations of primers and probes for each target together with the amplification conditions are listed in S2 Table. All samples were tested in triplicate on the CFX96 Real-Time System (Bio-Rad Laboratories Inc., Hercules, CA, United States of America).

We determined the analytical sensitivity of both assays[32] by replicate testing of a 7-dilution series of plasmid standards ranging from 1–1000 copies per reaction, under similar experimental conditions. We determined the analytical specificity of both assays by testing a collection of related bacterial and viral reference strains (S3 Table).

We added an exogenous internal amplification control plasmid (IAC) [33] into the bacterial multiplex assay in order to assess the quality of CSF sample extraction and the efficiency of amplification. We spiked varying concentrations of IAC into CSF and compared cycle threshold (Cq) values of each bacterial target to those obtained in the absence of the IAC.

### Study setting and patient selection

The Red Cross War Memorial Children’s Hospital (RCWMCH) is a 273-bed tertiary level paediatric hospital in Cape Town, Western Cape province, South Africa. The hospital provides emergency, general paediatric, specialised paediatric, paediatric surgical, and intensive care facilities and serves as a referral centre for the Western Cape and surrounding provinces. RCWMCH admits approximately 20 000 children per year, most of whom originate from poor peri-urban communities in the Western Cape. Children with suspected meningitis presenting to public sector primary care facilities within its drainage area are likely to be referred to RCWMCH for investigation and management.

We collected residual CSF samples from consecutive children aged between 60 days and 12 years, who presented to the acute care or outpatient departments of the RWMCH between 1 November 2012 and 31 October 2013, who had a lumbar puncture performed according to the attending clinician’s decision and whose CSF showed an abnormal cell count, defined as the presence of any neutrophils or > 5 lymphocytes/mm^3^. Patients with prior head trauma or ventricular peritoneal shunt (VP shunts) were excluded.

### Ethics

The study was approved by the Human Research Ethics Committee of the Faculty of Health Sciences, University of Cape Town, Cape Town, South Africa (HREC REF: 739/2013). The need for written informed consent from the participants was waived as testing was performed on residual CSF samples collected as part of routine clinical investigation. Results of the investigational tests were not made available to clinicians. Limited clinical information was collected by retrospective record review as part of a related study (HREC REF: 223/2015) using a standardised case record form. The requirement for written informed consent from the participants was also waived for this study. Specifically for use in this study we obtained basic demographic data, the final clinical diagnosis documented at discharge, and whether antibiotics were administered prior to lumbar puncture.

### Sample processing

CSF samples were stored at 4°C until completion of routine laboratory processing (typically 2–7 days to allow for after-requests by clinicians). Thereafter residual CSF samples were aliquoted into sterile 2ml cryogenic vials for storage at -80°C pending nucleic acid extraction.

### Conventional laboratory microbiology testing

Conventional laboratory testing was carried out at the National Health Laboratory Service (NHLS) diagnostic laboratories serving RCWMCH. Cerebro-spinal glucose and protein concentrations were measured on the Beckman Coulter AU480 automated analyser using the hexokinase G-6-PDH enzymatic method and colorimetric method respectively. Microscopy including differential white cell count, and Gram stain was performed according to standard protocols. For bacterial culture blood agar and boiled blood agar plates were inoculated and incubated in 5% CO^2^ at 35°C for up to 72 hours. Conventional ‘ ín house’ endpoint PCR for enterovirus[34], herpes simplex virus 1 and 2[35] and mumps virus[36] was performed if specifically requested by the clinician. Laboratory staff did not have access to detailed clinical information (apart from what was indicated on the laboratory request form) nor to results of index multiplex tests.

### CSF total nucleic acid extraction

Total nucleic acid was extracted from 400μl of CSF with the QIAsymphony virus/bacterial DSP kit (QIAGEN, Valencia, CA) using the QIAsymphony SP (QIAGEN, Valencia, CA) automated platform. Total nucleic acid was eluted in 60μl of elution buffer and stored at -21°C. Specimens with smaller available starting volumes (a minimum volume of 140μl) were topped up with ATL lysis buffer (QIAGEN, Valencia, CA) before extraction. An exogenous plasmid control was spiked into each sample at 200 copies/extraction volume as an extraction and internal amplification control (IAC). A volume of nuclease free water was included as the extraction negative control.

### Testing of CSF samples with multiplex real-time PCR assays

CSF samples were tested with the bacterial and viral multiplex RT-PCR assays as described above. For both assays a positive result was defined as a Cq value ≤ 35 and a negative result as a Cq value > 35 or no amplification. Extracted nuclease-free water was included as a no-template negative control. For each individual 96-well plate, there were 4 negative controls placed strategically amongst the samples. For the positive controls, 200 copies/ml of each of the 3 bacterial targets, plus the IAC, were co-amplified in a single well, and 200 copies/ml of each of the 3 viral targets were co-amplified in a second single well. All samples were tested in triplicate on the CFX96 Real-Time System (Bio-Rad Laboratories Inc., Hercules, CA, United States of America). The results of conventional microbiology tests were available to the researcher performing the multiplex RT-PCR assays.

### Data analysis

For the PCR optimization, the analytical sensitivity of the bacterial and viral multiplex assays was expressed as the lowest limit of detection (LOD) and was calculated using probit regression analysis (StatsDirect version 2.02). The mean Cq, standard deviation (SD) and coefficient of variation (CV) were expressed for the tested replicates of bacterial and viral multiplex assays.

Assuming a 50% increase in number of cases detected with the bacterial RT-PCR and based on previous CSF culture numbers, we calculated that a sample size of 375 was needed to provide 95% confidence intervals of 85.9% and 100% around a point estimate of sensitivity of 100%.

We assumed that the clinician’s decision to perform a lumbar puncture, together with the presence of an abnormal CSF, constituted a proxy for a suspected case of meningitis. Sensitivity and specificity of the bacterial RT-PCR were calculated, first using CSF culture as the reference standard and secondly using the. definitions of the World Health Organisation (WHO) Coordinated Invasive Bacterial Vaccine Preventable Diseases (IB-VPD) Surveillance Network[37], which classified patients as having suspected, probably or confirmed bacterial meningitis. Using this WHO definition, cases were classified as confirmed bacterial meningitis by culture or by identification with Gram stain of a bacterial pathogen (*S*. *pneumoniae*, *N*. *meningitidis* and *H*. *influenzae*) in the CSF or blood of a suspect case. Data was analysed with 2 x 2 tables, with calculation of 95% confidence intervals using the Wilson score method[38].

The results of the viral RT-PCR were compared to the results of in-house viral PCR testing [34–36] and viral culture, where available. The results of both viral and bacterial RT-PCR assays were compared to the final diagnosis at discharge as recorded in the patient’s clinical records.

## Results

### Development, optimisation and validation of the multiplex assays

#### Optimisation of the viral multiplex PCR

The viral singleplex assays were successfully multiplexed with comparable performance of both the singleplex and multiplex assays (S4 Table). The primers and probes of the viral targets showed minimal interference upon multiplexing. No cross-reactivity was observed between each primer and probe set, as seen by the lack of amplification of the other viral targets. The tested replicates on the viral multiplex assay showed good reproducibility and repeatability for all three targets resulting in low intra-assay and inter-assay CVs of less than 2% (S4 Table).

#### Analytical sensitivity and specificity

The lowest limit of detection for the bacterial multiplex was 2 copies/reaction for *S*. *pneumoniae* and 1 copy/reaction for both *H*. *influenzae* and *N*. *meningitidis*. (S5 Table). The viral multiplex detected 2 copies/reaction for both the herpes simplex virus and mumps virus. For enterovirus detection, the LOD was 3 copies/reaction (S6 Table). The threshold Cq value of 35, which was subsequently used for the sample testing corresponded to 10^1^ copies of DNA for each of the purified plasmid standards. The regression curve analysis for both multiplex assays showed linearity over a span of the different concentrations of the plasmid standards (S1 and S2 Figs). No amplification was observed on testing of the non-target bacterial and viral reference strains.

#### Incorporation of an internal amplification control into the bacterial multiplex assay

Incorporation of the IAC at a concentration of 200 copies/reaction together with 10 copies/reaction of each bacterial target had no significant effect on the Cq values of each bacterial target (S3 Fig and S7 Table).

### Multiplex RT PCR test results

**Fig 1:**
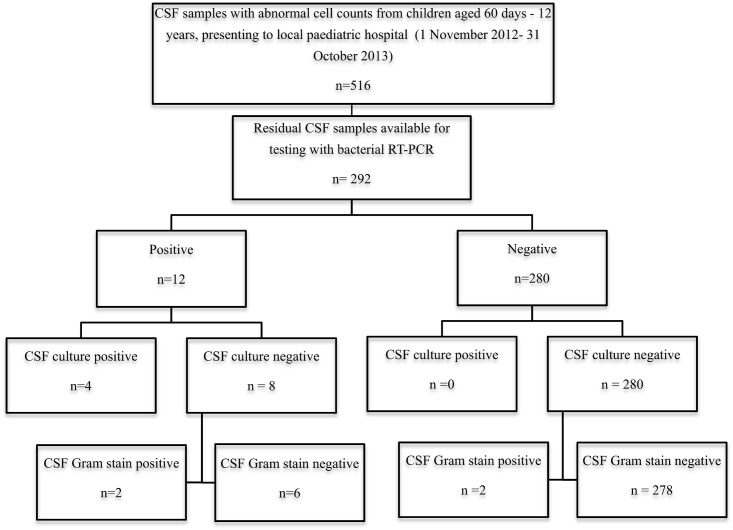
Flowchart showing results of bacterial multiplex realtime-PCR compared to cerebrospinal fluid microscopy and culture.

**Fig 2:**
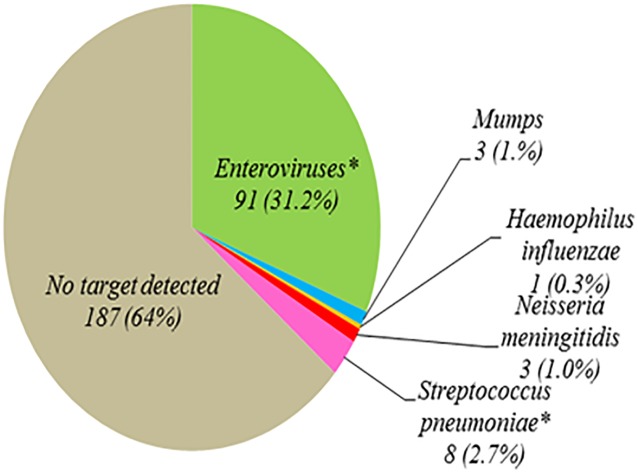
Results of bacterial and viral multiplex realtime PCR assays in cases of suspected meningitis in children presenting to the acute care or outpatient departments of the Red Cross War Memorial Children’s Hospital in Cape Town, South Africa, November 2012–October 2013 (n = 292).

**Fig 3:**
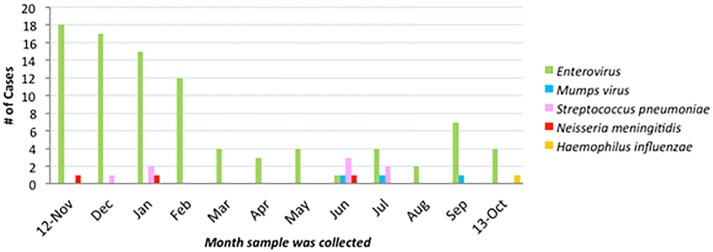
Monthly variation in detection of selected pathogens using bacterial and viral multiplex realtime PCR assays, in cases of suspected meningitis in children presenting to the acute care or outpatient departments of the Red Cross War Memorial Children’s Hospital in Cape Town, South Africa, November 2012–October 2013 (n = 292).

Over the 12 month period 3236 CSF samples from children at RCWMCH were submitted for microbiological testing. Of these 516 met the inclusion criteria for the study and sufficient residual CSF was available for testing with the multiplex RT PCR assays in 292 patients (Fig 1). The median age of the children was 35 months (IQR 2–144) and 52.6% were male. Bacterial target DNA was detected in 12 samples (4.1%) and viral target nucleic acids in 94 samples (32%), Fig 2. Herpes simplex was not detected in any sample. Both the internal amplification control (IAC) and positive controls amplified in all cases although there was some evidence of relative PCR inhibition in some samples (median Cq value for IAC for samples in which bacterial or viral target was identified = 28.5, range = 27.6–34.7). No amplification of negative controls was observed. Enterovirus was mainly detected in the summer months between November and February (Fig 3), whereas bacterial pathogens were detected sporadically in small numbers (1–4 cases/ month) in seven months of the year.

#### Comparison of bacterial RT-PCR assay with routine microbiology results

Bacterial pathogens were cultured from CSF in 4 samples, comprising *S*. *pneumoniae* (3 cases) and *H*. *influenzae* (1 case). No other bacterial causes of meningitis such as Gram-negative bacilli or listeria were isolated, although *Mycobacterium tuberculosis* complex was subsequently isolated from CSF in 3 children.

Using the WHO definition, ten cases were classified as confirmed bacterial meningitis: 4 on the basis of a positive CSF culture, 4 on the basis of a positive CSF Gram stain and 2 on the basis of the isolation of a potential pathogen from blood in the setting of an abnormal cerebrospinal fluid cell count. (Tables 1 and 2). Of the ten cases, bacterial target DNA was detected in 6 samples, including the 4 culture positive samples and two of the cases with a positive CSF Gram stain (Fig 1). The diagnosis in these 2 latter cases was supported by a positive blood culture in one, and a very high CSF polymorph cell count of 2480 in the other. In all cases the organism identified with RT-PCR was congruent with the CSF culture or CSF Gram stain morphology.

**Table 1:** Laboratory results of 10 cases classified as confirmed cases of bacterial meningitis according to the definitions of the World Health Organisation (WHO) Coordinated Invasive Bacterial Vaccine Preventable Diseases (IB-VPD) Surveillance Network.

Study number	RT-PCR ^a^ target	IAC ^b^Cq ^c^value	Bacterial RT-PCR Cq value^a^^c^	Polymorphs	Lymphocytes	Erythrocytes	Glucose	Protein	CSF^d^ Gram stain	CSF ^d^culture	Blood culture Gram stain	Blood culture culture
CSF Culture positive cases												
20	*S*. *pneumoniae*	33.1	33.1	10	2	2	37.8	99	GPDC^e^	*S*. *pneumoniae*	GPC^g^ in pairs	*S*. *pneumoniae*
30	*H*. *influenzae*	27.6	23.2	1754	614	26	5.4	112	GNB^f^	*H*. *influenzae*	GNB^f^	*H*. *influenzae*
112	*S*. *pneumoniae*	28.2	16.6	1620	60	25	63	82	GPDC^e^	*S*. *pneumoniae*	GPC^g^ in pairs	*S*. *pneumoniae*
293	*S*. *pneumoniae*	35.2	13.3	4500	800	0	0	200	GPC^g^ in chains	*S*. *pneumoniae*	No bacteria observed	No growth after 5 days
CSF Culture negative Gram stain positive cases												
114	*S*. *pneumoniae*	32.2	22.5	4500	800	0	63	48	GPC^g^	No growth after 3 days	No bacteria observed	No growth after 5 days
86	*N*. *meningitidis*	26.6	21.0	1680	800	400	5.4	102	GNDC^h^	No growth after 3 days	GNDC^h^	*N*. *meningitidis*
78	negative	25.0	-	0	5	55	66.6	12	GPDC^e^	No growth after 3 days	No bacteria observed	No growth after 5 days
277	(enterovirus)	38.0	(enterovirus 31.9)	255	170	20	64.8	25	GPDC^e^	No growth after 3 days	Gram-positive bacilli	*Bacillus* species
CSF Gram stain and CSF culture negative, blood culture positive												
98	negative	29.3	-	570	465	20	66.6	133	No bacteria observed	No growth after 3 days	Gram-positive cocci in chains	*S*. *pneumoniae*
164	negative	31.4	-	34	14	10 000	72	44	No bacteria observed	No growth after 3 days	Gram-positive cocci in chains	*S*. *pneumoniae*

^a^RT-PCR Real-time polymerase chain reaction,

^b^IAC internal amplification control,

^c^Cq cycle threshold,

^d^CSF cerebrospinal fluid,

^e^GDPC Gram-positive diplococci,

^f^GNB Gram-negative bacilli,

^g^GPC Gram-positive cocci,

^h^GNDC Gram-negative diplococci

**Table 2:** Clinical history and CRP results of 10 cases classified as confirmed cases of bacterial meningitis according to the definitions of the World Health Organisation (WHO) Coordinated Invasive Bacterial Vaccine Preventable Diseases (IB-VPD) Surveillance Network.

Study number	RT-PCR ^a^ target	Prior antibiotics	Discharge diagnosis	CRP^b^	Clinical history
CSF Culture positive cases					
20	*S*. *pneumoniae*	Nd^c^	Bacterial meningitis	34.3	
30	*H*. *influenzae*	No	Bacterial meningitis	52.3	
112	*S*. *pneumoniae*	Yes	Bacterial meningitis	102	
293	*S*. *pneumoniae*	No	Bacterial meningitis	389.5	
CSF Culture negative Gram stain positive cases					
114	*S*. *pneumoniae*	Yes	Bacterial meningitis	-	
86	*N*. *meningitidis*	No	Bacterial meningitis	244.9	
78	negative	No	Bacterial meningitis	4	5 months old, discharged after 2 days to continue daily ceftriaxone for total of 10 days
277	(enterovirus)^d^	Yes (IV ceftriaxone)	Bacterial meningitis	2.8	6 months old, symptoms of fever, diarrhoea and vomiting, transferred next day to another hospital to complete treatment, no subsequent re-admission
CSF Gram stain and CSF culture negative, blood culture positive					
98	negative	Yes (IV^e^ ceftriaxone)	Bacterial meningitis	224	HIV positive. Delay of at least 12 hours before lumbar puncture performed.
164	negative	No	Bacterial meningitis	17	7 months old, symptoms of fever, vomiting and seizures. Initially discharged, but re-called for treatment when blood culture result available

^a^RT-PCR Real-time polymerase chain reaction

^b^ CRP C-reactive protein

^c^nd not documented

^d^ enterovirus detected on viral RT-PCR assay

^e^IV intravenous

Four of the WHO confirmed cases had negative bacterial RT-PCR results: two with a positive CSF Gram stain (but negative cultures) and two with *S*. *pneumoniae* cultured from blood only. Of the two with positive CSF Gram stains (Gram-positive diplococci reported in both) there was no supporting microbiological evidence for a diagnosis of bacterial meningitis in one child, and in the other, enterovirus was detected with the viral RT-PCR while CRP was 2.8 mg/L. The first of these children was discharged after 2 days to continue outpatient parenteral antibiotic therapy. Of the two with *S*. *pneumoniae* isolated from blood only, one was an HIV positive child with a high CSF cell count and high CRP in whom the initial lumbar puncture was delayed for at least 12 hours after administration of antibiotics. The other child with *S*. *pneumoniae* bloodstream infection (and CRP of 17 mg/L) was well enough to be discharged from the acute care ward within 48 hours of admission and had to be recalled for treatment once the culture result became available.

In addition, the bacterial RT-PCR detected 6 cases that were not confirmed by WHO criteria. Details of these cases are presented in Tables 3 and 4. Three had a discharge diagnosis of bacterial meningitis, while the remaining 3 patients were diagnosed as viral meningitis, gastro-enteritis and upper respiratory tract infection respectively. Two of the unconfirmed cases had a CSF cell count >100 and 3 (of 4 in whom this documentation was available) had received antibiotics prior to lumbar puncture.

**Table 3:** Laboratory results of 6 cases in which bacterial target DNA was detected with bacterial multiplex realtime- PCR, but which were not confirmed according to the definitions of the World Health Organisation (WHO) Coordinated Invasive Bacterial Vaccine Preventable Diseases (IB-VPD) Surveillance Network.

Study number	RT-PCR ^a^target	IAC ^b^Cq ^c^value	Bacterial RT-PCR Cq value^a^ ^c^	Poly-morphs	Lymph-ocytes	Erythro-cytes	Glucose	Protein	CSF ^d^ Gram stain	CSF ^d^ culture	Blood culture Gram stain	Blood culture culture
177	*N*. *meningitidis*	28.3	30.1	11	240	25	57.6	71	Negative	No growth after 3 days	Negative	No growth after 5 days
250	*N*. *meningitidis*	34.3	34.8	0	14	14	54	18	Negative	No growth after 3 days	Negative	No growth after 5 days
228	*S*. *pneumoniae*	34.7	32.9	18	1	960	115.2	112	Negative	No growth after 3 days	Negative	No growth after 5 days
99	*S*. *pneumoniae* and enterovirus^e^	33.1	31.7 (*S*.*pneumoniae*) 34.6 (enterovirus)	95	36	310	70.2	38	Negative	No growth after 3 days	Negative	No growth after 5 days
296	*S*. *pneumoniae*	35.4	34.9	1	9	390	79.2	22	Negative	No growth after 3 days	Not taken	Not taken
8	*S*. *pneumoniae*	31.5	34.5	1	1	0	70.2	14	Negative	No growth after 3 days	Negative	No growth after 5 days

^a^RT-PCR Real-time polymerase chain reaction,

^b^IAC internal amplification control,

^c^Cq cycle threshold,

^d^CSF cerebrospinal fluid,

^e^ both *S*. *pneumoniae* and enterovirus were detected in this sample.

**Table 4:** Clinical history and CRP results of 6 cases in which bacterial target DNA was detected with bacterial multiplex realtime- PCR, but which were not confirmed according to the definitions of the World Health Organisation (WHO) Coordinated Invasive Bacterial Vaccine Preventable Diseases (IB-VPD) Surveillance Network.

Study number	RT-PCR^a^ target	Prior antibiotics	Discharge diagnosis	CRP^b^	Clinical history
177	*N*. *meningitidis*	Yes (IV^d^ ceftriaxone)	Bacterial meningitis	26	7 year old, lumbar puncture delayed 12 hrs after admission, discharged 10 days later, no subsequent re-admission, no rash documented
250	*N*. *meningitidis*	Yes (IM^e^ ceftriaxone)	Viral meningitis	13.7	Received at least 2 days additional antibiotics in hospital before discharge, no subsequent re-admission, no rash documented
228	*S*. *pneumoniae*	Yes	Bacterial meningitis	77	3 month old with underlying biliary atresia
99	*S*. *pneumoniae* and *enterovirus*	Not documented	Bacterial meningitis	1.0	18 month old, after 1 day transferred to another hospital to complete treatment, no subsequent re-admission
296	*S*. *pneumoniae*	No	Gastro-enteritis	1.0	Treated in hospital with antibiotics for 8 days, re-admitted 6 weeks later with pneumonia and investigated for tuberculosis (results negative)
8	*S*. *pneumoniae*	Not documented	URTI^c^	1.7	Discharged after 1 day, no subsequent re-admission

^a^RT-PCR Real-time polymerase chain reaction

^b^ CRP C-reactive protein

^c^ URTI upper respiratory tract infection

^d^IV intravenous

^e^ IM intramuscular

The Cq values for the bacterial target of the confirmed cases were significantly lower than the Cq values of the unconfirmed cases, with a median of 21.7 versus 33.7 (p = 0.01). The three patients with a discharge diagnosis other than bacterial meningitis had the highest Cq values (≥ 34.5) of all patients with a positive bacterial PCR.

Using CSF culture as a reference standard, the sensitivity and specificity of the bacterial RT-PCR were 100% (95% CI 51.0% -100%) and 97.2% (95% CI 94.6%– 98.6%) respectively, while compared to the WHO IB-VPD Surveillance Network definition of laboratory confirmed cases, the sensitivity and specificity were 60% (95% CI 31.3%– 83.2%) and 97.9% (95% CI 95.4% -99.0%) respectively. The positive and negative predictive values are shown in Table 5. The agreement between bacterial RT PCR and CSF culture was 97.3% (284/292), and between bacterial RT PCR and WHO definition of laboratory confirmed bacterial meningitis 96.6% (282/294).

**Table 5:** Sensitivity and specificity of bacterial multiplex realtime-PCR for the diagnosis of bacterial meningitis compared to CSF culture and compared to laboratory-confirmed bacterial meningitis cases according to the definitions of the World Health Organisation (WHO) Coordinated Invasive Bacterial Vaccine Preventable Diseases (IB-VPD) Surveillance Network[37].

		CSF culture		
		Positive	negative	total
Bacterial multiplex realtime PCR	positive	4	8	12
	negative	0	280	280
total		4	288	292
Test performance	%, (95% confidence intervals)			
Sensitivity	100% (51.0%– 100%)			
Specificity	97.2% (94.6% -98.6%)			
Positive predictive value	33.3% (13.8% - 60.9%)			
Negative predictive value	100% (98.7%– 100%)			
		Laboratory confirmed bacterial meningitis according to the definitions of World Health Organisation (WHO) Coordinated Invasive Bacterial Vaccine Preventable Diseases (IB-VPD) Surveillance Network.		
		Positive	negative	total
Bacterial multiplex realtime PCR	positive	6	6	12
	negative	4	276	280
total		10	282	292
Test performance	%, (95% confidence intervals)			
Sensitivity	60.0% (31.3% -83.2%)			
Specificity	97.9% (95.4% -99.0%)			
Positive predictive value	50.0% (25.4%– 74.6%)			
Negative predictive value	98.4% (96.4%– 99.4%)			

#### Comparison of viral RT-PCR assay with routine virology results

Details of the 94 cases detected by viral RT-PCR are provided in S8 Table. None of the cases positive by viral RT-PCR had a bacterial cause confirmed on CSF culture, though enterovirus was detected in one patient who was classified as having confirmed bacterial meningitis according to WHO criteria based solely on a CSF Gram stain showing Gram-positive diplococci. Enterovirus was also detected in an unconfirmed bacterial multiplex RT-PCR positive case (dual detection with S. pneumoniae).

Based on clinician request, 9 samples had undergone in-house viral PCR testing and 1 had been referred for viral culture (S9 Table). No requests were received for mumps virus PCR. There were no discrepancies between these results and the RT-PCR testing.

#### Comparison with the discharge diagnosis

Table 6 summarises the results of the 2 multiplex RT-PCR assays according to the discharge diagnosis, which was based on information derived from patient record review. Of the 292 suspected cases, 90 (30.8%) had a discharge diagnosis of bacterial meningitis or partially treated bacterial meningitis, while 97 (33.2%) had a discharge diagnosis of viral meningitis. Close to half of the patients in the categories of bacterial, viral and partially treated bacterial meningitis (50.7%, 45.3%, and 36.8% respectively) tested positive with the viral multiplex RT-PCR. Only 9/90 or 10.0% of patients diagnosed as bacterial meningitis or partially treated bacterial meningitis tested positive with the bacterial RT-PCR.

**Table 6:** Analysis of multiplex RT-PCR results according to discharge diagnosis.

Discharge Diagnosis		Multiplex RT-PCR results		
		Viral	Bacterial	Negative
Bacterial meningitis	71/292# (24.3%)	36 (50.7%)	9 (12.7%)	27 (38.0%)
Viral meningitis	97/292 (33.2%)	44 (45.3%)	1 (1.0%)	52 (53.6%)
Partially treated meningitis	19/292 (6.5%)	7 (36.8%)	-	12 (63.2%)
Other*	105/292 (36.0%)	7 (6.7%)	2 (0.02%)	96 (91.4%)

*the diagnosis includes other infections or non-infectious conditions or is not specified or unknown

^#^ 1 patient with a discharge diagnosis of bacterial meningitis had both S. pneumoniae and enterovirus detected and is included in both viral and bacterial categories

Comparison of the CSF findings showed significantly higher polymorphonuclear and lymphocyte cell counts in the bacterial and viral multiplex-positive groups compared those in whom no pathogen was detected: median CSF polymorphonuclear cell counts of 56.5, 40.5 and 2/mm3 respectively (p < 0.05) and median CSF lymphocyte counts 48, 42.5 and 9/mm3 respectively(p < 0.05). There was a significant difference in CSF protein concentration between the bacterial positive group (0.77g/l) and the viral and no pathogen groups (0.28 g/l and 0.25 g/l respectively) (p < 0.05). There were no differences in median red blood cell count or glucose concentration.

## Discussion

In this population of children presenting to a tertiary hospital and undergoing lumbar puncture which revealed abnormal CSF cell counts, the use of a bacterial and a viral multiplex RT-PCR together targeting 6 commonest pathogens gave promising results. Bacterial target DNA was detected in 4.1% (12/292) of samples and viral target nucleic acids in 32% (94/292). The sensitivity and specificity of the bacterial RT-PCR were 100% (95% CI 51–100%) and 97.2% (95% CI 94.6–98.6%) respectively, compared to CSF culture, while the results of the viral RT-PCR compared favourably with the conventional virology results available in a small number of cases. CSF cell counts were significantly higher in the bacterial and viral multiplex-positive groups compared those in whom no pathogen was detected, while CSF protein was raised in the bacterial multiplex-positive group only. Only 10% of children with a discharge diagnosis of bacterial or partially treated meningitis tested positive with the bacterial multiplex-PCR while nearly a half of patients with a discharge diagnosis of bacterial, viral or partially treated bacterial meningitis tested positive with the viral multiplex RT-PCR.

The 6 organisms targeted (*S*. *pneumoniae*, *N*. *meningitidis*, *H*. *influenzae* type b, enteroviruses, mumps virus, herpes simplex virus) were chosen since they are the most common causes of bacterial and viral meningitis in children beyond the neonatal period in our local South African setting, and in some neighbouring countries [26] [39, 40]. In addition, enteroviruses [41] and mumps virus [42] may present with a florid pleocytosis and neutrophil predominance in CSF that can cause diagnostic confusion. HSV though rare as a cause of isolated meningitis, is critical for detection in cases of meningo-encephalitis where early empiric treatment with acyclovir is recommended. Availability of a RT-PCR would improve turn-around time compared to the current in-house end-point PCR.

The proportions of bacteria and viruses detected by RT-PCR were consistent with previously described local epidemiology, including the increased prevalence of enterovirus over the summer months [21] [7, 26]. Very low numbers of bacterial meningitis cases were detected in the study, either by conventional or molecular testing. This may be partly due to the decreasing burden of disease due to *S*. *pneumoniae* and *H*. *influenzae* type b following implementation of successful vaccination programmes targeting these pathogens in 2009 (pneumococcal polysaccharide-protein conjugate vaccine (PCV)) and 1999 (H. influenzae type b (Hib))[25] [22] respectively. The low number of confirmed cases also suggests a very low threshold for the performance of lumbar puncture in this paediatric population, most of whom will have been referred from primary health care level. For practical reasons we based inclusion in the study on the presence of an abnormal CSF cell count, rather than on clinical suspicion of meningitis. It is therefore possible that in some children LPs were performed for other reasons, e.g. investigation of epilepsy. This might have artificially reduced the prevalence rate of bacterial or viral meningitis. We also did not adjust the CSF white cell count for the presence of red blood cells. This could have resulted in the inclusion of cases not meeting the definition of suspect cases, thereby contributing to low prevalence of bacterial meningitis.

The predominance of viral meningitis, comprising 32.2% of cases, was not unexpected, as viral meningitis is the more common though frequently unrecognised cause of meningitis [7, 43]. Diagnostic tests for viral causes of meningitis are not performed routinely in our setting, because of cost as well as delayed turn-around time and the labour-intensive nature of viral culture and conventional PCR. Clinicians rarely request viral diagnostic testing, perhaps for these reasons or perhaps because of a lack of awareness [4]. We were therefore only able to compare our viral RT-PCR results with the in-house end-point PCR in a small minority of cases (9 patients), but no discrepancies were noted.

Based on the clinicians’ records 36% of children were considered to have a final discharge diagnosis other than bacterial or viral meningitis. (This category included other infections as well as non-infectious conditions or unknown or non-specified conditions.) Negative RT-PCR results were obtained in 91.4% of children in this category. These observations would be in keeping with local clinical practice where exclusion of the diagnosis of meningitis is an important consideration.

The calculated sensitivity and specificity of the bacterial RT-PCR compared to CSF culture were 100% (95% CI 51.0% -100%) and 97.2% (95% CI 94.6%– 98.6%) respectively and compared to WHO laboratory- confirmed cases 60.0% (95% CI 31.3% -83.2%) and 97.9% (95% CI 95.4%– 99.0%) respectively. Unfortunately the confidence intervals for sensitivity were very wide due to small numbers of positives. There was complete agreement in organism identification between the bacterial RT-PCR and CSF culture.

Discrepancies between the bacterial RT-PCR and conventional microbiology results may have been due to limitations in the conventional microbiology reference methods or in the bacterial RT-PCR method. It is difficult to validate a new, potentially more sensitive test method when no perfect ‘gold standard’ or reference method is available. CSF culture may have limited sensitivity, especially if antibiotics have been administered prior to lumbar puncture. In our study 6 samples testing positive with the bacterial RT-PCR were not confirmed with conventional microbiological testing, but 3 of the 4 in whom this information was recorded had received antibiotics prior to lumbar puncture. The sensitivity of Gram stain is less affected by prior antibiotic therapy [11, 44], though it may be reduced if the delay to lumbar puncture is prolonged > 24 hours [45]. However, the specificity of Gram stain, being dependent on operator performance, is also imperfect. Other studies have reported specificities of 98%- 99% [11, 46]. In our study where Gram stain was performed in a busy routine diagnostic service, isolated false positive results may occur. Potentially false positive Gram stain results seem likely in two patients in whom the diagnosis of bacterial meningitis was not supported by other microbiological or clinical evidence, one of whom had enterovirus detected on viral RT-PCR. This could account for the lower sensitivity of bacterial RT-PCR when using the WHO definitions of laboratory- confirmed cases as a reference standard. Since the RT-PCRs in this study were batched and performed retrospectively, we were unable to confirm the accuracy of these discrepant Gram stains.Latent class analysis (LCA) modelling is a method that combines results of multiple diagnostic tests in a statistical model to generate estimates of disease prevalence while avoiding any assumptions about the accuracy of particular tests. Using a LCA model, in patients with suspected meningitis, Lu et al showed that the sensitivity of culture was inferior to that of bacterial multiplex RT-PCR or Gram stain [11].

False positive and false negative results can nevertheless occur in molecular diagnostic tests. False positives may be due to lack of specificity of primers or probes used, or due to contamination during specimen collection or laboratory testing. Traumatic or ‘bloody’ taps which are relatively common in this hospital can also introduce organisms present in blood into CSF. Given that 3 of the unconfirmed bacterial RT-PCR-positive cases had a discharge diagnosis other than bacterial meningitis and had very high Cq values (≥ 34.5) suggests that it might be prudent to retest specimens with Cq values ≥ 34.0 or ≥ 34.5 to exclude contamination.

Common causes of false negatives are limited analytical or clinical sensitivity, inhibition (potentially due to presence of blood due to a traumatic tap), failure of amplification or detection due to unrecognised variation in target of interest, sampling errors (e.g. due to testing of very small volumes), degradation of nucleic acids (especially RNA) or failure to test for the causative pathogen [32, 47, 48]. In this study inclusion of an IAC in every sample minimised the possibility of inhibition, but the wide range of IAC Cq values for samples in which bacterial or viral target was identified (27.6–34.7) suggests that a degree of inhibition could have occurred in some samples. Ideally a larger prospective study that included careful review of Gram stain results, as well as routine use of conventional viral diagnostic tests, is required to fully resolve the issues of sensitivity and specificity.

The purpose of this test if introduced into routine practice would be to supplement other diagnostic tests, with the potential for more rapid and more sensitive results. Gram stain because of its immediacy, and culture because of its broad range for detection of pathogens not included in the multiplex assay, must both still be carried out[11]. Given the serious nature of bacterial meningitis and consequences of lack of treatment, the results of the multiplex assays would have to be interpreted in light of the pre-test probability of disease and according to the clinician’s judgement. The PPV and NPV for the bacterial multiplex assay compared to CSF culture were 33.3% and 100% respectively (Table 5), but the high NPV may be due to the low prevalence of disease in the population, and the low PPV due to the insensitivity of culture in patients with prior antibiotic therapy.

Detection of a viral or bacterial cause of meningitis confirms the diagnosis and thus reduces the need for further investigations. Detection of a specific bacterial cause may potentially reduce duration of antibiotic therapy depending on the organism identified, while detection of a viral cause permits discontinuation of empiric antibiotics. A negative result in the bacterial assay may in certain circumstances prompt discontinuation of antibiotics. This is likely to be particularly valuable in cases of prior antibiotic use where fears of false negative culture results may lead to a tendency to over treat.

While the RT-PCR assays included in the study had limited coverage compared to some of the commercial assays, it is likely that these assays have the potential to identify the majority of meningitis cases at a much lower cost [19] [18]. Although currently RT-PCR assays are typically performed in large centralised laboratories, future developments in point of care testing may facilitate the devolution of testing to smaller hospitals.

The potential for reduction of unnecessary antibiotic therapy is shown by the fact that 50% of patients with a discharge diagnosis of bacterial meningitis actually had a viral pathogen detected on RT-PCR. In addition, bacterial meningitis is usually treated with a lengthy course of 7–10 days of broad spectrum intravenous antibiotics (ceftriaxone) and therefore more targeted diagnosis would be a major gain for antibiotic stewardship. Additional benefits include reduced hospitalisation, with attendant potential for reduction in hospital-acquired infections, as well as more accurate and more rapid data for a public health response where indicated, and more complete surveillance data[17, 49].

The assays could also be applied to an adult population where issues of prior antibiotic therapy and lack of diagnostic tests for viral meningitis are similar concerns. However, since adults with meningitis tend to present more commonly with typical clinical features as compared to children, the pretest probability of meningitis is likely to be higher and molecular testing more cost-effective, although enteroviral meningitis is much less common in adults outside of an outbreak setting.

In summary these two multiplex RT-PCR assays are useful additional tests with a rapid turn-around time that could assist in the diagnosis of community acquired meningitis in both children and adults and could support antibiotic stewardship efforts.

## Supporting information

S1 Table Primers and probes for the bacterial and viral multiplex real-time PCR assays. (DOCX)

S2 Table Reaction conditions for the bacterial and viral multiplex real-time PCR assays. (DOCX)

S3 Table Related bacterial and viral reference strains. (DOCX)

S4 Table Repeatability and reproducibility of the optimised viral multiplex real time PCR assay. (DOCX)

S5 Table Limit of detection for the bacterial multiplex realtime PCR assay. (DOCX)

S6 Table Limit of detection viral multiplex realtime PCR assay. (DOCX)

S7 Table Incorporation of an internal amplification control into the bacterial multiplex assay. (DOCX)

S8 Table Clinical and laboratory details for the viral realtime-PCR positive cases (n = 94). (DOCX)

S9 Table Results of viral multiplex realtime-PCR compared to routine viral diagnostic testing (when requested by clinician). (DOCX)

S1 Fig Standard curves for the individual bacterial target amplification in the bacterial multiplex real-time PCR assay. (DOCX)

S2 Fig Standard curves for the individual viral target amplification in the viral multiplex real-time PCR assay. (DOCX)

S3 Fig Incorporation of an internal amplification control into the bacterial multiplex assay showing impact on amplification of bacterial targets. (DOCX)

S1 File Appendix A: Bacterial and viral strains used as reference standards. Appendix B: Primers used for amplification of cloning inserts for the target genes. Appendix C: PCR assay conditions for amplification of the cloning inserts for the target genes.
(DOCX)

S2 File De-identified raw data. (XLSX)
