# Supplementary material for: Diagnostic accuracy of two multiplex real-time polymerase chain reaction assays for the diagnosis of meningitis in children in a resource-limited setting
Source: PLoS One. 2017 Mar 27;12(3):e0173948. doi: 10.1371/journal.pone.0173948 (PMC5367690; doi:10.1371/journal.pone.0173948)
Supplement: S1 Table — (DOCX) [file pone.0173948.s001.docx]

S1Table: Primers and probes for the bacterial and viral multiplex real-time PCR assays

| **Target** | **Primers** | **Primer sequence (5'---- 3')** | **Gene target**  **and size** | **Reference** |
| --- | --- | --- | --- | --- |
| *S. pneumoniae* | F373 | ACGCAATCTAGCAGCTGAAGCA | *LytA* gene  75 bp | 28 |
|  | R424 | TCGTGCGTTTTAATTCCAGCT |  |  |
|  | Pb400 | TxRd -TGCCGAAAACGC"T"TGATACAGGGAG-BHQ2dT* |  |  |
| *H. influenzae* | HPDF729 | AGATTGGAAAGAAACACAAGAAAAAGA | *Hpd* gene  113 bp | 28 |
|  | HPDR819 | CACCATCGGCATATTTAACCACT |  |  |
|  | PBR762 | Cy5 -AAACATCCAATCG"T"AATTATAGTTTACCCAATAACCCBHQ2 dT* |  |  |
| *N. meningitidis* | F753 | 5′-TGTGTTCCGCTATACGCCATT-3′ | *CtrA* gene  114 bp | 28 |
|  | R846 | 5′-GCCATATTCACACGATATACC-3′ |  |  |
|  | Pb820 | FAM -AACCTTGAGCAA"T"CCATTTATCCTGACGTTCT-BHQ1dT* |  |  |
| Herpes simplex | HSV-F | 5′-CATCACCGACCCGGAGAGGGAC | UL30 gene  92 bp | 31 |
|  | HSV-R | 5′-GGGCCAGGCGCTTGTTGGTGTA |  |  |
|  | Probe | Cy5 -CCGCCGAACTGAGCAGACACCCGCGC-BHQ2 |  |  |
| Enterovirus | Ev-F | 5′-CCTGAATGCGGCTAATCC-3′ | 5' UTR region  144 bp | 8, 29 |
|  | Ev-R | 5′-ATTGTCACCATAAGCAGCC-3′ |  |  |
|  | Probe | TxRd -ACCGACTACTTTGGGTGTCCGTGTTTC- BHQ2 |  |  |
| Mumps | F1073 | 5′-TCTCACCCATAGCAGGGAGTTATAT | Fusion protein gene 79 bp | 30 |
|  | R1151 | 5′-GTTAGACTTCGACAGTTTGCAACAA |  |  |
|  | Probe | FAM -AGGCGATTTGTAGCACTGGATGGAACA –BHQ1 |  |  |

TxRd- Texas Red; Cy5- Cyanine 5; FAM - 6-Flourescein; BHQ- Black Hole Quencher; *- the quenchers are internally placed at the “T” base region.
