# Supplementary material for: Diagnostic accuracy of two multiplex real-time polymerase chain reaction assays for the diagnosis of meningitis in children in a resource-limited setting
Source: PLoS One. 2017 Mar 27;12(3):e0173948. doi: 10.1371/journal.pone.0173948 (PMC5367690; doi:10.1371/journal.pone.0173948)
Supplement: S2 Table — (DOCX) [file pone.0173948.s002.docx]

S2 Table: Reaction conditions for the bacterial and viral multiplex real-time PCR assays

| **Multiplex Targets** | **Primers and probes** | **Reaction concentration** | **Initial denaturation** | **PCR Cycles** |
| --- | --- | --- | --- | --- |
| ***S. pneumoniae***  ***H. influenzae***  ***N. meningitidis***  ***IAC**** | *S. pneumoniae-F* | 200nM | 50^o^C (2mins)95^o^C (10mins) | 95^o^C (15s) 60^o^C(60s)* (X40) |
|  | *S. pneumoniae-R* | 200nM |  |  |
|  | *S. pneumoniae Probe* | 200nM |  |  |
|  | *H. influenzae-F* | 300nM |  |  |
|  | *H. influenzae-R* | 100nM |  |  |
|  | *H. influenzae Probe* | 200nM |  |  |
|  | *N. meningitidis-F* | 300nM |  |  |
|  | *N. meningitidis-R* | 900nM |  |  |
|  | *N. meningitidis Probe* | 100nM |  |  |
|  | *IAC-F* | 200nM |  |  |
|  | *IAC-R* | 200nM |  |  |
|  | *IAC Probe* | 200nM |  |  |
| ***Herpes simplex***  ***Enterovirus***  ***Mumps*** | *Herpes simplex -F* | 500nM | 50^o^C (10mins) 95^o^C (5mins) | 95^o^C (15s) 62^o^C(30s)* (X45) |
|  | *Herpes simplex -R* | 500nM |  |  |
|  | *Herpes simplex Probe* | 200nM |  |  |
|  | *Enterovirus-F* | 700nM |  |  |
|  | *Enterovirus-R* | 700nM |  |  |
|  | *Enterovirus Probe* | 200nM |  |  |
|  | *Mumps -F* | 300nM |  |  |
|  | *Mumps -R* | 300nM |  |  |
|  | *Mumps Probe* | 200nM |  |  |

F- Forward primer; R- Reverse primer; IAC- internal amplification control

*IAC only included in final stages/final version of bacterial multiplex assay
