# Supplementary material for: Diagnostic accuracy of two multiplex real-time polymerase chain reaction assays for the diagnosis of meningitis in children in a resource-limited setting
Source: PLoS One. 2017 Mar 27;12(3):e0173948. doi: 10.1371/journal.pone.0173948 (PMC5367690; doi:10.1371/journal.pone.0173948)
Supplement: S3 Table — (DOCX) [file pone.0173948.s003.docx]

S3 Table: Related bacterial and viral reference strains

| **Test organism** | **Characteristics** | **Reference** |
| --- | --- | --- |
| **Bacteria** | | |
| *Acinetobacter baumannii* | Clinical isolate | UCT |
| *Enterobacter cloacae* | Clinical isolate | UCT |
| *Enterococcus faecalis ATCC 51299* | Type strain | UCT |
| *Escherichia coli ATCC 25922* | Type strain | UCT |
| *Haemophilus influenzae ATCC 49247* | Type strain | NICD |
| *Klebsiella pneumoniae ATCC 1705* | Type strain | UCT |
| *Klebsiella pneumoniae ATCC 1706* | Type strain | UCT |
| *Klebsiella pneumoniae ATCC 700603* | Type strain | UCT |
| *Neisseria meningitidis EMGM6* | Type strain | NICD |
| *Pseudomonas aeruginosa ATCC 27853* | Type strain | UCT |
| *Pseudomonas aeruginosa ATCC 27853* | Type strain | UCT |
| *Staphylococcus scuri* | Clinical isolate | UCT |
| *Serratia marcescens* | Clinical isolate | UCT |
| *Staphylococcus aureus* | Clinical isolate | UCT |
| *Staphylococcus epidermidis* | Clinical isolate | UCT |
| *Staphylococcus saprophyticus* | Clinical isolate | UCT |
| *Streptococcus anginosus ATCC 2008* | Type strain | NICD |
| *Streptococcus bovis ATCC 9809* | Type strain | NICD |
| *Streptococcus pneumoniae ATCC 496190* | Type strain | NICD/GSH |
| *Streptococcus pyogenes* | Clinical isolate | GSH |
| *Streptococcus thermophilus ATCC 192583* | Type strain | NICD |
| *Streptococcus viridans* | Clinical isolate | GSH |
| *Streptococcus viridans ATCC 7868* | Type strain | NICD |
| *Streptococcus pyogenes ATCC19615* | Type strain | NICD |
| **Viruses** | | |
| Cytomegalovirus | Clinical isolate | GSH |
| Epstein Barr virus | Clinical isolate | GSH |
| Varicella zoster virus | Clinical isolate | GSH |
| Human herpes virus 6 | Clinical isolate | GSH |
| Rhinovirus | Clinical isolate | GSH |
| Parainfluenza 2 | Clinical isolate | GSH |
| Human respiratory syncytial virus b | Clinical isolate | GSH |
| Mumps | Clinical isolate | NICD |
| Enterovirus (echovirus 13, 24, 30) | Clinical isolate | NICD |
| Herpes simplex subtype 1, and 2 | Clinical isolate | NICD |

GSH- Groote Schuur Hospital; NICD- National Institute for Communicable Diseases; UCT- University of Cape Town.
