# Supplementary material for: Diagnostic accuracy of two multiplex real-time polymerase chain reaction assays for the diagnosis of meningitis in children in a resource-limited setting
Source: PLoS One. 2017 Mar 27;12(3):e0173948. doi: 10.1371/journal.pone.0173948 (PMC5367690; doi:10.1371/journal.pone.0173948)
Supplement: S4 Table — (DOCX) [file pone.0173948.s004.docx]

S4 Table: Repeatability and reproducibility of the optimised viral multiplex real time PCR assay.

| **Intra-assay variations** | | |  | **Inter-assay variations** | | | |
| --- | --- | --- | --- | --- | --- | --- | --- |
| Mean  (Cq value) | Standard deviation (SD) | % Coefficient of variation (CV) |  | Mean  (Cq value) | Standard  deviation (SD) | % Coefficient of variation (CV) | |
| **Multiplex real time PCR targeting mumps viral RNA (4ng/**µ**l)** | | | | | | | |
| S- 25.30 | 0.118 | 0.46 |  | M1-25.94 | 0.15 | | 0.59 |
| M- 25.97 | 0.159 | 0.61 |  | M2- 25.97 | 0.16 | | 0.61 |
|  |  |  |  | M3- 27.15 | 0.40 | | 1.48 |
| **Multiplex real time PCR targeting enterovirus viral RNA (2ng/**µ**l)** | | | | | | | |
| S- 28.32 | 0.071 | 0.25 |  | M1-29.07 | 0.04 | | 0.14 |
| M- 28.20 | 0.006 | 0.02 |  | M2- 28.20 | 0.006 | | 0.02 |
|  |  |  |  | M3- 27.48 | 0.34 | | 1.23 |
| **Multiplex real time PCR targeting herpes simplex viral DNA (1.7ng/**µ**l)** | | | | | | | |
| S- 19.21 | 0.075 | 0.39 |  | M1- 18.09 | 0.10 | | 0.58 |
| M- 19.17 | 0.060 | 0.31 |  | M2- 19.17 | 0.06 | | 0.31 |
|  |  |  |  | M3- 14.10 | 0.04 | | 0.33 |

Note: Mean Cq values mentioned indicates the average of 3 replicates of same run (intra-assay) and between runs (inter-assay); M1-M3, multiplex runs; S, singleplex.
