# Supplementary material for: Diagnostic accuracy of two multiplex real-time polymerase chain reaction assays for the diagnosis of meningitis in children in a resource-limited setting
Source: PLoS One. 2017 Mar 27;12(3):e0173948. doi: 10.1371/journal.pone.0173948 (PMC5367690; doi:10.1371/journal.pone.0173948)
Supplement: S5 Table — (DOCX) [file pone.0173948.s005.docx]

S5 Table: Limit of detection for the bacterial multiplex realtime PCR assay

|  | **Plasmid copies** | **No. of replicates** | **No. positive** | **% Positive** | **Cq Mean** | **Cq Std. Dev** | **%CV** |
| --- | --- | --- | --- | --- | --- | --- | --- |
| *Streptococcus pneumoniae* | 1000 | 8 | 8 | 100 | 27.15 | 0.122 | 0.448 |
|  | 500 | 8 | 8 | 100 | 28.02 | 0.093 | 0.331 |
|  | 200 | 8 | 8 | 100 | 30.00 | 0.097 | 0.322 |
|  | 100 | 8 | 8 | 100 | 30.47 | 0.102 | 0.333 |
|  | 50 | 8 | 8 | 100 | 31.57 | 0.204 | 0.647 |
|  | 10 | 8 | 8 | 100 | 33.92 | 0.480 | 1.415 |
|  | 1 | 8 | 6 | 75 | 36.37 | 0.618 | 1.698 |
|  | **Plasmid copies** | **No. of replicates** | **No. positive** | **% Positive** | **Cq Mean** | **Cq Std. Dev** | **%CV** |
| *Neisseria meningitidis* | 1000 | 8 | 8 | 100 | 26.91 | 0.132 | 0.489 |
|  | 500 | 8 | 8 | 100 | 27.76 | 0.113 | 0.407 |
|  | 200 | 8 | 8 | 100 | 29.05 | 0.054 | 0.186 |
|  | 100 | 8 | 8 | 100 | 30.07 | 0.065 | 0.217 |
|  | 50 | 8 | 8 | 100 | 30.91 | 0.221 | 0.716 |
|  | 10 | 8 | 8 | 100 | 33.05 | 0.391 | 1.182 |
|  | 1 | 8 | 8 | 100 | 36.74 | 0.720 | 1.959 |
|  | **Plasmid copies** | **No. of replicates** | **No. positive** | **% Positive** | **Cq Mean** | **Cq Std. Dev** | **%CV** |
| *Haemophilus influenzae* | 1000 | 8 | 8 | 100 | 26.12 | 0.152 | 0.581 |
|  | 500 | 8 | 8 | 100 | 26.99 | 0.073 | 0.272 |
|  | 200 | 8 | 8 | 100 | 28.04 | 0.054 | 0.191 |
|  | 100 | 8 | 8 | 100 | 28.89 | 0.125 | 0.431 |
|  | 50 | 8 | 8 | 100 | 29.66 | 0.137 | 0.463 |
|  | 10 | 8 | 8 | 100 | 31.90 | 0.377 | 1.182 |
|  | 1 | 8 | 8 | 100 | 35.01 | 0.409 | 1.170 |
