# Supplementary material for: Diagnostic accuracy of two multiplex real-time polymerase chain reaction assays for the diagnosis of meningitis in children in a resource-limited setting
Source: PLoS One. 2017 Mar 27;12(3):e0173948. doi: 10.1371/journal.pone.0173948 (PMC5367690; doi:10.1371/journal.pone.0173948)
Supplement: S6 Table — (DOCX) [file pone.0173948.s006.docx]

S6 Table: Limit of detection viral multiplex realtime PCR assay

|  | **Plasmid copies** | **No. of replicates** | **% Positive** | **Cq Mean** | **Cq Std. Dev** | **%CV** |
| --- | --- | --- | --- | --- | --- | --- |
| enterovirus | 1000 | 8 | 100 | 26.98 | 0.103 | 0.381 |
|  | 500 | 8 | 100 | 27.92 | 0.159 | 0.570 |
|  | 200 | 8 | 100 | 29.02 | 0.136 | 0.468 |
|  | 100 | 8 | 100 | 30.00 | 0.260 | 0.867 |
|  | 50 | 8 | 100 | 30.66 | 0.479 | 1.563 |
|  | 10 | 8 | 100 | 32.66 | 0.352 | 1.076 |
|  | 1 | 8 | 50 | 34.66 | 0.893 | 2.578 |
|  | **Plasmid copies** | **No. of replicates** | **% Positive** | **Cq Mean** | **Cq Std. Dev** | **%CV** |
| mumps | 1000 | 8 | 100 | 27.22 | 0.097 | 0.356 |
|  | 500 | 8 | 100 | 28.22 | 0.143 | 0.508 |
|  | 200 | 8 | 100 | 29.48 | 0.161 | 0.545 |
|  | 100 | 8 | 100 | 30.60 | 0.306 | 1.000 |
|  | 50 | 8 | 100 | 31.31 | 0.250 | 0.797 |
|  | 10 | 8 | 100 | 33.85 | 0.573 | 1.693 |
|  | 1 | 8 | 87.5 | 36.68 | 0.693 | 1.889 |
|  | **Plasmid copies** | **No. of replicates** | **% Positive** | **Cq Mean** | **Cq Std. Dev** | **%CV** |
| herpes simplex | 1000 | 8 | 100 | 25.69 | 0.115 | 0.448 |
|  | 500 | 8 | 100 | 26.47 | 0.230 | 0.868 |
|  | 200 | 8 | 100 | 27.72 | 0.226 | 0.816 |
|  | 100 | 8 | 100 | 28.61 | 0.145 | 0.506 |
|  | 50 | 8 | 100 | 29.66 | 0.220 | 0.742 |
|  | 10 | 8 | 100 | 32.12 | 0.276 | 0.860 |
|  | 1 | 8 | 87.5 | 35.09 | 0.505 | 1.441 |
