# Supplementary material for: Diagnostic accuracy of two multiplex real-time polymerase chain reaction assays for the diagnosis of meningitis in children in a resource-limited setting
Source: PLoS One. 2017 Mar 27;12(3):e0173948. doi: 10.1371/journal.pone.0173948 (PMC5367690; doi:10.1371/journal.pone.0173948)
Supplement: S7 Table — (DOCX) [file pone.0173948.s007.docx]

S7 Table: Incorporation of an Internal Amplification Control into the bacterial multiplex assay

| **Flourophore** | **Sample** | **Cq Mean** | **Cq Std. Dev** | **95 %CI** |
| --- | --- | --- | --- | --- |
| Cy5 | Bacterial multiplex RT-PCR + *H.influenzae* plasmid | 32.65 | 0.631 | (31.41-33.88) |
| Cy5 | Bacterial multiplex RT-PCR + *H.influenzae* plasmid + IAC primers and probes | 33.81 | 0.653 | (32.53-35.09) |
| Cy5 | Bacterial multiplex RT-PCR + *H.influenzae* plasmid + IAC primers and probes + IAC plasmid | 32.93 | 0.735 | (31.49-34.37) |
| VIC | IAC plasmid + primers and probes for bacterial targets | 31.52 | 0.498 | (30.54-32.50) |
| VIC | IAC plasmid + primers and probes for bacterial targets + IAC primers and probes | 31.87 | 0.495 | (30.90-32.84) |
| VIC | IAC plasmid + primers and probes for bacterial targets + IAC primers and probes + *H.influenzae* | 31.99 | 0.403 | (31.20-32.78) |
| Texas Red | Bacterial multiplex RT-PCR + *S. pneumonia* plasmid | 31.77 | 0.448 | (29.23-31.41) |
| Texas Red | Bacterial multiplex RT-PCR + *S. pneumonia* plasmid + IAC primers and probes | 31.94 | 0.760 | (30.62-32.08) |
| Texas Red | Bacterial multiplex RT-PCR + *S. pneumonia* plasmid + IAC primers and probes + IAC plasmid | 31.98 | 0.427 | (30.09-32.05) |
| VIC | IAC_ triplex | 30.32 | 0.558 | (29.23-31.41) |
| VIC | IAC_quadruplex | 31.35 | 0.374 | (30.62-32.09) |
| VIC | IAC + *S. pneumoniae* | 31.07 | 0.498 | (30.09-32.05) |
| FAM | Bacterial multiplex RT-PCR + *N. meningitides* plasmid | 23.99 | 0.102 | (23.79-24.19) |
| FAM | Bacterial multiplex RT-PCR + *N. meningitides* plasmid + IAC primers and probes | 23.91 | 0.114 | (23.69-24.13) |
| FAM | Bacterial multiplex RT-PCR + *N. meningitides* plasmid + IAC primers and probes + IAC plasmid | 23.73 | 0.072 | (23.59-23.87) |
| VIC | IAC_singleplex | 32.05 | 0.707 | (30.66-33.44) |
| VIC | IAC_quadruplex | 32.03 | 0.274 | (31.49-32.57) |
| VIC | IAC + *N. meningitidis* | 30.62 | 0.801 | (29.05-32.19) |

IAC- internal amplification control; Cy5 - Cyanine 5; FAM - 6-Flourescein; Std. Dev- standard deviation; CI- confidence interval
