# Supplementary material for: Diagnostic accuracy of two multiplex real-time polymerase chain reaction assays for the diagnosis of meningitis in children in a resource-limited setting
Source: PLoS One. 2017 Mar 27;12(3):e0173948. doi: 10.1371/journal.pone.0173948 (PMC5367690; doi:10.1371/journal.pone.0173948)
Supplement: S8 Table — (DOCX) [file pone.0173948.s008.docx]

S8 Table: Clinical and laboratory details for the viral realtime-PCR positive cases (n=94)

| **Study number** | **RT-PCR Results** | **DC Rx** | **DC Dx** | **Polymorphs** | **Lymphocytes** | **Erythrocytes** | **Glucose mg/dl** | **Protein mg/dl** | **Gram stain** | **CSF Culture** | **BC gram stain** | **BC results** | **In-house viral PCR** |
| --- | --- | --- | --- | --- | --- | --- | --- | --- | --- | --- | --- | --- | --- |
| **56** | *Mumps virus* | Antibiotics | VM | 10 | 675 | 0 | 75.6 | 29 | Negative | Neg after 3days | - | - | CSF HSV 1 + 2 negative |
| **100** | *Mumps virus* | Antibiotics | Other | 5 | 900 | 15 | 57.6 | 48 | Negative | Neg after 3days | Negative | Neg after 3days | - |
| **410** | *Mumps virus* | Antibiotics | VM | 44 | 139 | 9 | 79.2 | 33 | Negative | Neg after 3days | Negative | Neg after 3days | - |
| **5** | *Enterovirus* | Antibiotics | VM | 63 | 13 | 6 | 50.4 | 23 | Negative | Neg after 3days | Negative | Neg after 3days | - |
| **10** | *Enterovirus* | Antibiotics | PTM | 105 | 675 | 5 | 59.4 | 45 | Negative | Neg after 3days | Negative | Neg after 3days | - |
| **11** | *Enterovirus* | ND | VM | 30 | 38 | 2 | 73.8 | 25 | Negative | Neg after 3days | - | - | - |
| **12** | *Enterovirus* | ND | VM | 59 | 69 | 240 | 54 | 19 | Negative | Neg after 3days | Negative | Neg after 3days | - |
| **15** | *Enterovirus* | Antibiotics | BM | 175 | 515 | 200 | 41.4 | 63 | Negative | Neg after 3days | Negative | Neg after 3days | - |
| **24** | *Enterovirus* | Antibiotics | BM | 38 | 143 | 14 | 39.6 | 77 | Negative | Neg after 3days | Negative | Other | CSF Enterovirus positive |
| **31** | *Enterovirus* | Antibiotics | BM | 52 | 39 | 0 | 70.2 | 28 | Negative | Neg after 3days | Negative | Neg after 3days | CSF Enterovirus positive |
| **35** | *Enterovirus* | Antibiotics | BM, PTM | 390 | 55 | 5 | 68.4 | 25 | Negative | Neg after 3days | Negative | Neg after 3days | - |
| **36** | *Enterovirus* | - | VM | 3 | 16 | 0 | 52.2 | 19 | Negative | Neg after 3days | - | - | - |
| **41** | *Enterovirus* | Antibiotics | PTM | 95 | 330 | 45 | 70.2 | 36 | Negative | Neg after 3days | - | - | - |
| **43** | *Enterovirus* | Antibiotics | BM | 615 | 185 | 20 | 75.6 | 42 | Negative | Neg after 3days | Negative | Neg after 3days | CSF Enterovirus positive |
| **45** | *Enterovirus* | - | VM | 2 | 5 | 0 | 59.4 | 31 | Negative | Neg after 3days | - | - | - |
| **47** | *Enterovirus* | Antibiotics | BM | 80 | 520 | 5 | 61.2 | 51 | Negative | Neg after 3days | Negative | Neg after 3days | - |
| **52** | *Enterovirus* | Antibiotics | BM | 115 | 33 | 0 | 66.6 | 32 | Negative | Neg after 3days | - | - | - |
| **60** | *Enterovirus* | Antibiotics | BM | 200 | 46 | 15 | 68.4 | 25 | Negative | Neg after 3days | Negative | CONS | - |
| **64** | *Enterovirus* | Antibiotics | BM | 185 | 60 | 3360 | 68.4 | 39 | Negative | Neg after 3days | Negative | Neg after 3days | - |

CSF- cerebrospinal fluid; RT-PCR- real-time PCR; ND - not documented; - -not taken, VM- viral meningitis; BC- blood culture; BM- bacterial meningitis; PTM- partially treated meningitis; GPDC- Gram positive diplococci; GPC- Gram positive cocci; GNB- Gram negative bacilli; GNDC- Gram negative diplococci; CONS- Coagulase Negative staphylococci; DC- Discharge; DC Rx- Discharge treatment; DC Dx- Discharge diagnosis

| **Study number** | **RT-PCR Results** | **DC Rx** | **DC Dx** | **Polymorphs** | **Lymphocytes** | **Erythrocytes** | **Glucose mg/dl** | **Protein mg/dl** | **Gram stain** | **CSF Culture** | **BC gram stain** | **BC results** | **In-house viral PCR** |
| --- | --- | --- | --- | --- | --- | --- | --- | --- | --- | --- | --- | --- | --- |
| **97** | *Enterovirus* | Antibiotics | BM | 141 | 14 | 5 | 70.2 | 23 | Negative | Neg after 3days | - | - | - |
| **99** | *S. pneumoniae & Enterovirus* | Antibiotics | BM | 95 | 36 | 310 | 70.2 | 38 | Negative | Neg after 3days | Negative | Neg after 3days | - |
| **400** | *Enterovirus* | Antibiotics | VM | 0 | 6 | 1480 | 84.6 | 29 | Negative | Neg after 3days | Negative | Neg after 3days | - |
| **121** | *Enterovirus* | Antibiotics | BM | 215 | 285 | 5 | 63 | 42 | Negative | Neg after 3days | Negative | Neg after 3days | - |
| **135** | *Enterovirus* | ND | VM | 133 | 407 | 10 | 72 | 0 | Negative | Neg after 3days | Negative | Neg after 3days | - |
| **143** | *Enterovirus* | ND | ND | 9 | 7 | 90 | 64.8 | 13 | Negative | Neg after 3days | - | - | - |
| **150** | *Enterovirus* | Antibiotics | BM | 83 | 110 | 30 | 63 | 24 | Negative | Neg after 3days | - | - | - |
| **154** | *Enterovirus* | ND | VM | 116 | 145 | 7 | 70.2 | 29 | Negative | Neg after 3days | - | - | - |
| **165** | *Enterovirus* | Other/ URTI DC on panado | Other | 2 | 1 | 675 | 68.4 | 24 | Negative | Neg after 3days | - | - | - |
| **176** | *Enterovirus* | Antibiotics | VM | 240 | 51 | 170 | 68.4 | 37 | Negative | Neg after 3days | Negative | Neg after 3days | - |
| **178** | *Enterovirus* | ND | VM | 0 | 28 | 0 | 55.8 | 30 | Negative | Neg after 3days | - | - | - |
| **182** | *Enterovirus* | Antibiotics | PTM | 86 | 28 | 260 | 61.2 | 27 | Negative | Neg after 3days | Negative | Neg after 3days | - |
| **185** | *Enterovirus* | Antibiotics | VM | 6 | 105 | 1 | 64.8 | 33 | Negative | Neg after 3days | - | - | - |
| **186** | *Enterovirus* | Antibiotics | VM | 4 | 6 | 15 | 70.2 | 16 | Negative | Neg after 3days | - | - | - |
| **187** | *Enterovirus* | Antibiotics | VM | 595 | 35 | 0 | 88.2 | 33 | Negative | Neg after 3days | - | - | - |
| **188** | *Enterovirus* | DC on panado and brufen | VM | 4 | 22 | 22 | 59.4 | 19 | Negative | Neg after 3days | Negative | Neg after 3days | - |
| **191** | *Enterovirus* | - | VM | 2 | 3 | 160 | 57.6 | 20 | Negative | Neg after 3days | Negative | Neg after 3days | CSF Enterovirus positive |
| **192** | *Enterovirus* | Antibiotics | VM | 9 | 88 | 5 | 63 | 35 | Negative | Neg after 3days | Negative | Neg after 3days | - |

CSF- cerebrospinal fluid; RT-PCR- real-time PCR; ND - not documented; - -not taken, VM- viral meningitis; BC- blood culture; BM- bacterial meningitis; PTM- partially treated meningitis; GPDC- Gram positive diplococci; GPC- Gram positive cocci; GNB- Gram negative bacilli; GNDC- Gram negative diplococci; CONS- Coagulase Negative staphylococci; DC- Discharge; DC Rx- Discharge treatment; DC Dx- Discharge diagnosis; URTI- upper respiratory tract infection

| **Study number** | **RT-PCR Results** | **DC Rx** | **DC Dx** | **Polymorphs** | **Lymphocytes** | **Erythrocytes** | **Glucose mg/dl** | **Protein mg/dl** | **Gram stain** | **CSF Culture** | **BC gram stain** | **BC results** | **In-house viral PCR** |
| --- | --- | --- | --- | --- | --- | --- | --- | --- | --- | --- | --- | --- | --- |
| **195** | *Enterovirus* | DC on panado and brufen | BM | 1 | 3 | 155 | 72 | 24 | Negative | Neg after 3days | - | - | - |
| **196** | *Enterovirus* | Antibiotics | BM | 185 | 685 | 33 | 55.8 | 46 | Negative | Neg after 3days | Negative | Neg after 3days | - |
| **198** | *Enterovirus* | Antibiotics | PTM | 41 | 48 | 8 | 63 | 25 | Negative | Neg after 3days | Negative | Neg after 3days | - |
| **203** | *Enterovirus* | Antibiotics | VM | 107 | 15 | 1 | 68.4 | 33 | Negative | Neg after 3days | Negative | Neg after 3days | - |
| **204** | *Enterovirus* | Antibiotics, DC on panado | VM | 394 | 2880 | 72 | 52.2 | 81 | Negative | Neg after 3days | Negative | Neg after 3days | - |
| **206** | *Enterovirus* | Antibiotics | VM | 140 | 225 | 10 | 88.2 | 24 | Negative | Neg after 3days | Negative | Neg after 3days | - |
| **212** | *Enterovirus* | Antibiotics | BM | 365 | 60 | 0 | 59.4 | 26 | Negative | Neg after 3days | Negative | Neg after 3days | - |
| **213** | *Enterovirus* | Antibiotics | ND | 6 | 14 | 3440 | 55.8 | 28 | Negative | Neg after 3days | Negative | Neg after 3days | - |
| **214** | *Enterovirus* | Antibiotics | BM | 16 | 5 | 1 | 63 | 24 | Negative | Neg after 3days | Negative | Neg after 3days | - |
| **216** | *Enterovirus* | DC on panado | VM | 30 | 23 | 17 | 66.6 | 20 | Negative | Neg after 3days | Negative | Neg after 3days | - |
| **221** | *Enterovirus* | - | BM | 9 | 20 | 7040 | 52.2 | 44 | Negative | Neg after 3days | Negative | Neg after 3days | - |
| **222** | *Enterovirus* | Antibiotics | PTM | 15 | 53 | 1 | 66.6 | 19 | Negative | Neg after 3days | - | - | - |
| **224** | *Enterovirus* | Antibiotics | BM | 306 | 149 | 8 | 59.4 | 32 | Negative | Neg after 3days | - | - | - |
| **225** | *Enterovirus* | Antibiotics | BM | 13 | 109 | 25 | 50.4 | 103 | Negative | Neg after 3days | Negative | Neg after 3days | - |
| **232** | *Enterovirus* | DC on panado. | VM | 6 | 2 | 5 | 73.8 | 20 | Negative | Neg after 3days | Negative | Neg after 3days | - |
| **233** | *Enterovirus* | Antibiotics | VM, PTM | 3 | 9 | 0 | 73.8 | 24 | Negative | Neg after 3days | - | - | - |
| **234** | *Enterovirus* | Antibiotics | VM | 15 | 5 | 10000 | 77.4 | 22 | Negative | Neg after 3days | - | - | - |
| **237** | *Enterovirus* | - | VM | 3 | 9 | 1 | 55.8 | 32 | Negative | Neg after 3days | - | - | - |

CSF- cerebrospinal fluid; RT-PCR- real-time PCR; ND - not documented; - -not taken, VM- viral meningitis; BC- blood culture; BM- bacterial meningitis; PTM- partially treated meningitis; GPDC- Gram positive diplococci; GPC- Gram positive cocci; GNB- Gram negative bacilli; GNDC- Gram negative diplococci; CONS- Coagulase Negative staphylococci; DC- Discharge; DC Rx- Discharge treatment; DC Dx- Discharge diagnosis

| **Study number** | **RT-PCR Results** | **DC Rx** | **DC Dx** | **Polymorphs** | **Lymphocytes** | **Erythrocytes** | **Glucose mg/dl** | **Protein mg/dl** | **Gram stain** | **CSF Culture** | **BC gram stain** | **BC results** | **In-house viral PCR** |
| --- | --- | --- | --- | --- | --- | --- | --- | --- | --- | --- | --- | --- | --- |
| **238** | *Enterovirus* | Antibiotics | BM | 485 | 10 | 0 | 68.4 | 36 | Negative | Neg after 3days | Negative | Neg after 3days | - |
| **241** | *Enterovirus* | DC on panado | VM | 16 | 24 | 4 | 72 | 22 | Negative | Neg after 3days | - | - | - |
| **244** | *Enterovirus* | DC on panado | VM | 44 | 27 | 5 | 46.8 | 28 | Negative | Neg after 3days | - | - | - |
| **246** | *Enterovirus* | Antibiotics | BM | 120 | 87 | 64 | 81 | 44 | Negative | Neg after 3days | - | - | - |
| **247** | *Enterovirus* | Antibiotics | BM | 7 | 140 | 1 | 54 | 17 | Negative | Neg after 3days | - | - | - |
| **253** | *Enterovirus* | DC panado and folate | VM | 96 | 135 | 42 | 73.8 | 27 | Negative | Neg after 3days | - | - | - |
| **254** | *Enterovirus* | Antibiotics | PTM | 13 | 242 | 1205 | 59.4 | 34 | Negative | Neg after 3days | Negative | Neg after 3days | - |
| **256** | *Enterovirus* | - | VM | 2 | 8 | 2 | 55.8 | 14 | Negative | Neg after 3days | Negative | Neg after 3days | - |
| **257** | *Enterovirus* | Antibiotics | BM | 42 | 248 | 10720 | 64.8 | 52 | Negative | Neg after 3days | Negative | Neg after 3days | - |
| **260** | *Enterovirus* | Antibiotics | BM | 490 | 91 | 22 | 63 | 88 | Negative | Neg after 3days | - | - | - |
| **261** | *Enterovirus* | DC on panado | VM | 3 | 3 | 16 | 61.2 | 26 | Negative | Neg after 3days | - | - | - |
| **262** | *Enterovirus* | Antibiotics | VM | 12 | 0 | 4 | 59.4 | 22 | Negative | Neg after 3days | Negative | Neg after 3days | - |
| **265** | *Enterovirus* | - | VM | 29 | 3 | 19 | 64.8 | 21 | Negative | Neg after 3days | - | - | - |
| **267** | *Enterovirus* | DC on panado | VM | 60 | 75 | 150 | 64.8 | 25 | Negative | Neg after 3days | - | - | - |
| **268** | *Enterovirus* | Antibiotics | BM | 154 | 101 | 10 | 81 | 25 | Negative | Neg after 3days | GPC in clusters | CONS | - |
| **273** | *Enterovirus* | DC on panado | VM | 13 | 63 | 5 | 90 | 24 | Negative | Neg after 3days | - | - | - |
| **274** | *Enterovirus* | Antibiotics | BM | 74 | 85 | 7 | 59.4 | 20 | Negative | Neg after 3days | Negative | Neg after 3days | - |

CSF- cerebrospinal fluid; RT-PCR- real-time PCR; ND - not documented; - -not taken, VM- viral meningitis; BC- blood culture; BM- bacterial meningitis; PTM- partially treated meningitis; GPDC- Gram positive diplococci; GPC- Gram positive cocci; GNB- Gram negative bacilli; GNDC- Gram negative diplococci; CONS- Coagulase Negative staphylococci; DC- Discharge; DC Rx- Discharge treatment; DC Dx- Discharge diagnosis

| **Study number** | **RT-PCR Results** | **DC Rx** | **DC Dx** | **Polymorphs** | **Lymphocytes** | **Erythrocytes** | **Glucose mg/dl** | **Protein mg/dl** | **Gram stain** | **CSF Culture** | **BC gram stain** | **BC results** | **In-house viral PCR** |
| --- | --- | --- | --- | --- | --- | --- | --- | --- | --- | --- | --- | --- | --- |
| **276** | *Enterovirus* | ND | VM | 25 | 11 | 1640 | 68.4 | 27 | Negative | Neg after 3days | Negative | Neg after 3days | - |
| **277** | *Enterovirus* | Antibiotics | BM | 255 | 170 | 20 | 64.8 | 25 | GPDC | Neg after 3days | GPB | Bacillus species | - |
| **279** | *Enterovirus* | Antibiotics | BM | 403 | 29 | 4 | 73.8 | 32 | Negative | Neg after 3days | - | - | - |
| **280** | *Enterovirus* | DC on panado | VM | 32 | 50 | 3 | 59.4 | 29 | Negative | Neg after 3days | - | - | - |
| **286** | *Enterovirus* | Antibiotics | VM | 10 | 2 | 0 | 73.8 | 15 | Negative | Neg after 3days | Negative | Neg after 3days | - |
| **289** | *Enterovirus* | DC on panado | VM | 25 | 100 | 2 | 63 | 24 | Negative | Neg after 3days | Negative | Neg after 3days | - |
| **290** | *Enterovirus* | Other | ND | 18 | 139 | 5 | 64.8 | 29 | Negative | Neg after 3days | - | - | - |
| **291** | *Enterovirus* | Antibiotics | VM | 119 | 34 | 5 | 70.2 | 23 | Negative | Neg after 3days | Negative | Neg after 3days | - |
| **294** | *Enterovirus* | Antibiotics | BM | 159 | 58 | 21 | 66.6 | 26 | Negative | Neg after 3days | Negative | Neg after 3days | - |
| **309** | *Enterovirus* | Antibiotics | BM | 300 | 205 | 5 | 54 | 99 | Negative | Neg after 3days | Negative | Neg after 3days | - |
| **310** | *Enterovirus* | Other | BM | 32 | 6 | 4 | 73.8 | 29 | Negative | Neg after 3days | - | - | - |
| **312** | *Enterovirus* | Other | VM | 9 | 1 | 597 | 64.8 | 15 | Negative | Neg after 3days | Negative | Neg after 3days | - |
| **313** | *Enterovirus* | Other | VM | 76 | 72 | 5 | 54 | 45 | Negative | Neg after 3days | Negative | Neg after 3days | - |
| **315** | *Enterovirus* | Antibiotics | BM | 47 | 10 | 0 | 63 | 17 | Negative | Neg after 3days | Negative | Neg after 3days | - |
| **319** | *Enterovirus* | DC on panado | VM | 26 | 33 | 48 | 75.6 | 36 | Negative | Neg after 3days | Negative | Neg after 3days | - |
| **321** | *Enterovirus* | Antibiotics | BM | 214 | 33 | 2 | 55.8 | 48 | Negative | Neg after 3days | Negative | Neg after 3days | - |
| **324** | *Enterovirus* | Antibiotics | BM | 0 | 19 | 5 | 52.2 | 25 | Negative | Neg after 3days | - | - | - |
| **326** | *Enterovirus* | ND | VM | 165 | 225 | 350 | 64.8 | 42 | Negative | Neg after 3days | - | - |  |

CSF- cerebrospinal fluid; RT-PCR- real-time PCR; ND - not documented; - -not taken, VM- viral meningitis; BC- blood culture; BM- bacterial meningitis; PTM- partially treated meningitis; GPDC- Gram positive diplococci; GPC- Gram positive cocci; GNB- Gram negative bacilli; GNDC- Gram negative diplococci; CONS- Coagulase Negative staphylococci; DC- Discharge; DC Rx- Discharge treatment; DC Dx- Discharge diagnosis

| **Study number** | **RT-PCR Results** | **DC Rx** | **DC Dx** | **Polymorphs** | **Lymphocytes** | **Erythrocytes** | **Glucose mg/dl** | **Protein mg/dl** | **Gram stain** | **CSF Culture** | **BC gram stain** | **BC results** | **In-house viral PCR** |
| --- | --- | --- | --- | --- | --- | --- | --- | --- | --- | --- | --- | --- | --- |
| **413** | *Enterovirus* | Antibiotics | BM | 0 | 6 | 1 | 63 | 18 | Negative | Neg after 3days | GPC in chains, GPC in clusters | *Streptococcus sanguinis* and *Staphylococcus haemolyticus* | - |
| **501** | *Enterovirus* | ND | ND | 14 | 18 | 6 | 63 | 29 | Negative | Neg after 3days | - | - | - |
| **504** | *Enterovirus* | ND | ND | 2 | 7 | 3 | 73.8 | 22 | Negative | Neg after 3days | - | - | - |

CSF- cerebrospinal fluid; RT-PCR- real-time PCR; ND - not documented; - -not taken, VM- viral meningitis; BC- blood culture; BM- bacterial meningitis; PTM- partially treated meningitis; GPDC- Gram positive diplococci; GPC- Gram positive cocci; GNB- Gram negative bacilli; GNDC- Gram negative diplococci; CONS- Coagulase Negative staphylococci; DC- Discharge; DC Rx- Discharge treatment; DC Dx- Discharge diagnosis
