# Supplementary material for: Diagnostic accuracy of two multiplex real-time polymerase chain reaction assays for the diagnosis of meningitis in children in a resource-limited setting
Source: PLoS One. 2017 Mar 27;12(3):e0173948. doi: 10.1371/journal.pone.0173948 (PMC5367690; doi:10.1371/journal.pone.0173948)
Supplement: S9 Table — (DOCX) [file pone.0173948.s009.docx]

S9 Table: Results of viral multiplex realtime-PCR compared to routine viral diagnostic testing (when requested by clinician)

| **Study number** | **RT-PCR result** | | | **In-house PCR** | | | **viral culture** |
| --- | --- | --- | --- | --- | --- | --- | --- |
|  | **Enterovirus** | **Herpes simplex** | **Mumps** | **Enterovirus** | **Herpes simplex** | **Mumps** | - |
| **24** | positive | negative | negative | positive | - | - | - |
| **29** | negative | negative | negative | negative | - | - | - |
| **31** | positive | negative | negative | positive | - | - | - |
| **43** | positive | negative | negative | positive | - | - | - |
| **56** | negative | negative | positive | - | negative | - | - |
| **88** | negative | negative | negative | negative | negative | - | viral culture negative |
| **109** | negative | negative | negative | - | negative | - | - |
| **115** | negative | negative | negative | - | negative | - | - |
| **191** | positive | negative | negative | positive | - | - | - |

CSF- cerebrospinal fluid; RT-PCR- real-time PCR; - not tested
