# Supplementary material for: Diagnostic accuracy of two multiplex real-time polymerase chain reaction assays for the diagnosis of meningitis in children in a resource-limited setting
Source: PLoS One. 2017 Mar 27;12(3):e0173948. doi: 10.1371/journal.pone.0173948 (PMC5367690; doi:10.1371/journal.pone.0173948)
Supplement: S1 Fig — (DOCX) [file pone.0173948.s010.docx]

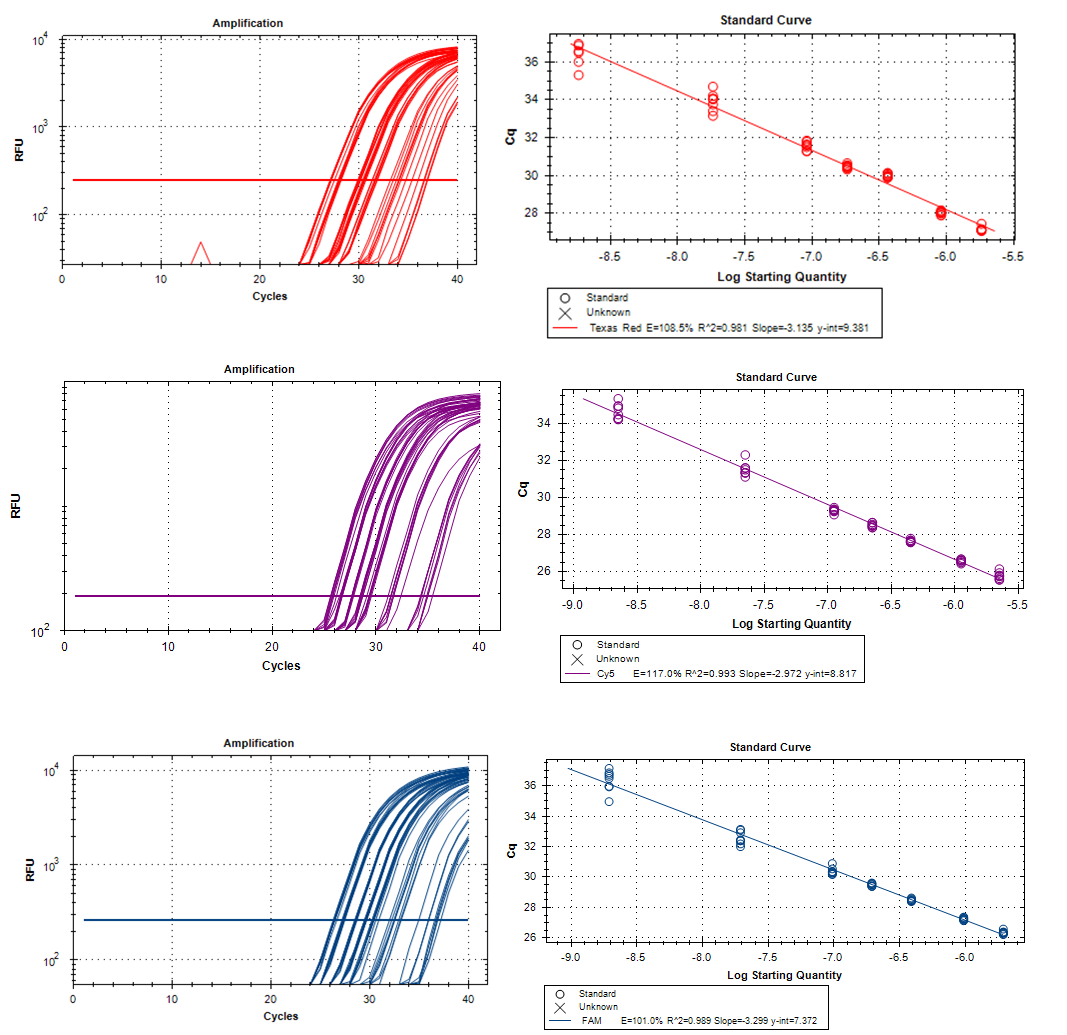


S1 Figure: Standard curves for the individual bacterial target amplification in the bacterial multiplex real-time PCR assay.

1. *S. pneumoniae*, B- *H. influenzae*, C- *N. meningitidis*.

Left: Amplification curves of the prepared plasmid standard concentrations (ranging from 1000

copies/reaction to 1copy/reaction);

Right-: Linearity of the tested replicates and efficiency of amplification of the standard.
