# Supplementary material for: Diagnostic accuracy of two multiplex real-time polymerase chain reaction assays for the diagnosis of meningitis in children in a resource-limited setting
Source: PLoS One. 2017 Mar 27;12(3):e0173948. doi: 10.1371/journal.pone.0173948 (PMC5367690; doi:10.1371/journal.pone.0173948)
Supplement: S2 Fig — (DOCX) [file pone.0173948.s011.docx]

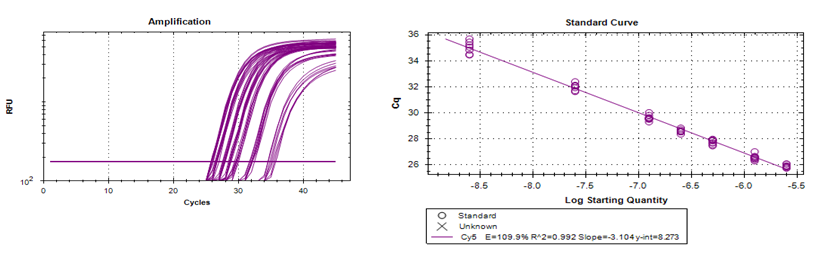


**A**


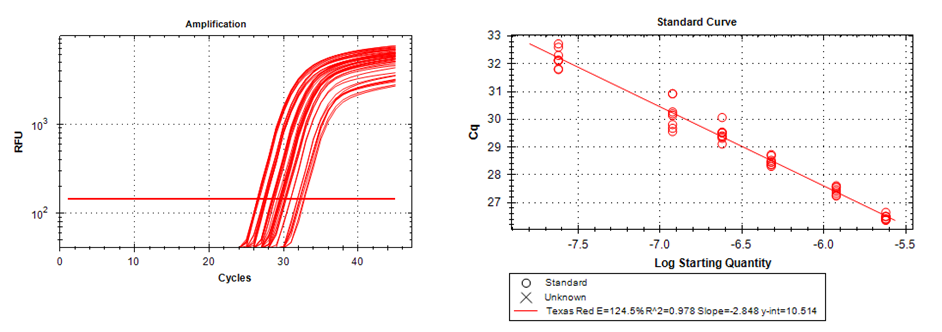


**B**


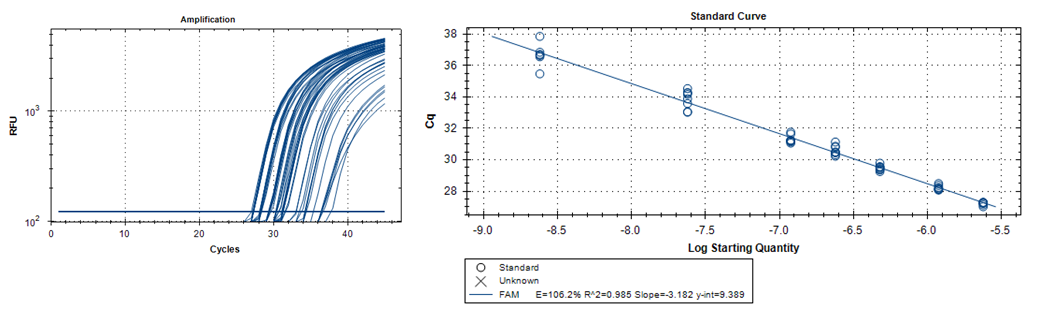


**C**

S2 Figure: Standard curves for the individual viral target amplification in the viral multiplex real-time PCR assay.

1. herpes simplex, B- enterovirus, C- mumps.

Left: Amplification curves of the prepared plasmid standard concentrations (ranging from 1000 copies/reaction to 1copy/reaction );

Right: Linearity of the tested replicates and efficiency of amplification of the standard.
