# Supplementary material for: Diagnostic accuracy of two multiplex real-time polymerase chain reaction assays for the diagnosis of meningitis in children in a resource-limited setting
Source: PLoS One. 2017 Mar 27;12(3):e0173948. doi: 10.1371/journal.pone.0173948 (PMC5367690; doi:10.1371/journal.pone.0173948)
Supplement: S3 Fig — (DOCX) [file pone.0173948.s012.docx]

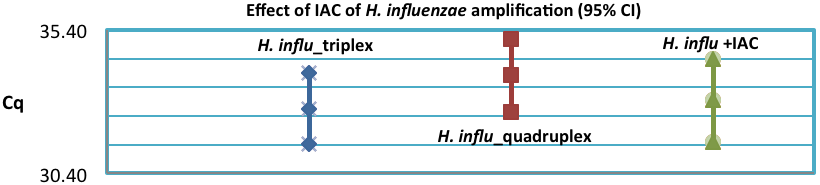


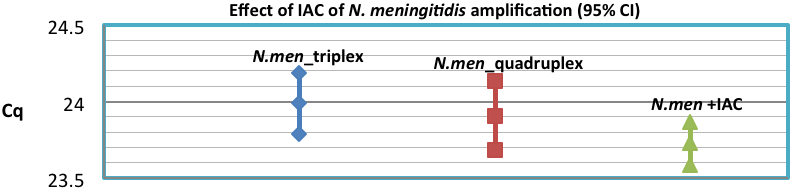


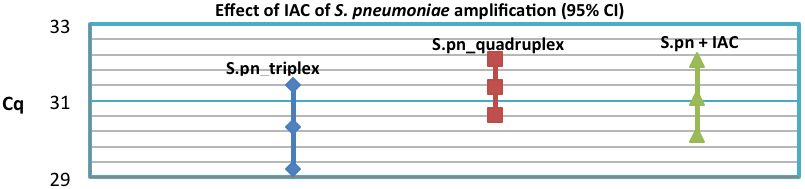


S.pn- *S. pneumoniae*; N.men- *N. meningitidis*; H.influ- *H. influenzae*; IAC- Internal amplification control CI- confidence interval

S3 Figure: Incorporation of an Internal Amplification Control into the bacterial multiplex assay showing impact on amplification of bacterial targets
