# Supplementary material for: Diagnostic accuracy of two multiplex real-time polymerase chain reaction assays for the diagnosis of meningitis in children in a resource-limited setting
Source: PLoS One. 2017 Mar 27;12(3):e0173948. doi: 10.1371/journal.pone.0173948 (PMC5367690; doi:10.1371/journal.pone.0173948)
Supplement: S1 File — Appendix A: Bacterial and viral strains used as reference standards. Appendix B: Primers used for amplification of cloning inserts for the target genes. Appendix C: PCR assay conditions for amplification of the cloning inserts for the target genes. (DOCX) [file pone.0173948.s013.docx]

S1 File. Appendix A: Bacterial and viral strains used as reference standards. Appendix B: Primers used for amplification of cloning inserts for the target genes. Appendix C : PCR assay conditions for amplification of the cloning inserts for the target genes.

Appendix A: Bacterial and viral strains used as reference standards

| Control strains | Characteristics | Source |
| --- | --- | --- |
| Bacterial strains | | |
| *Streptococcus pneumoniae* ATCC^a^ 496190 | Type strain of *S. pneumoniae* | NICD^b^ |
| *Neisseria meningitidis* EMGM ^c^6 | Type strain of *N. meningitidis* | NICD^b^ |
| *Haemophilus influenzae* ATCC^a^ 49247 | Type strain of *H. influenzae* | NICD^b^ |
| Viral strains | | |
| mumps | Clinical isolate | NICD^b^ |
| enterovirus (echovirus 13, 24, 30) | Clinical isolate | NICD^b^ |
| herpes simplex subtype 1, and 2 | Clinical isolate | NICD^b^ |

^a^ ATCC American Type Culture Collection, ^b^NICD National Institute for Communicable Diseases, ^c^ EMGM: European Monitoring Group on Meningococci.

Bacterial reference strains were cultured on 2% boiled blood agar (Greenpoint Media Laboratory, National Health Laboratory Service, Cape Town, South Africa), aerobically in 5% CO2 for 24 hrs. DNA was extracted using the Zymofungal/bacterial Miniprep DNA kit (ZymoResearch Corp., Irvine, United States of America), and quantified using the NanoDrop® ND-1000 (NanoDrop Technologies Inc., Wilmington, United States of America). Live viral cells (mumps virus, enterovirus and herpes simplex) were heat killed at 57°C for 1 hour and mechanically lysed at 50 Hz for 5 min in a tissue lyser (Qiagen, FRITSCH GmbH, Idar-Oberstein, Germany). The crude lysate was centrifuged 13 000 rpm for 10mins and 400µl of the supernatant was used for total nucleic acid extraction using the QIAsymphony® SP platform (Qiagen, Hilden, Germany) and the QIAsymphony® Virus/Bacteria Mini Kit (Qiagen, Hilden, Germany).

DNA extracted from the reference control strains was used to generate inserts for the targets of interest using PCR primers and conditions listed in Appendix B and C respectively.

Appendix B: Primers used for amplification of cloning inserts for the target genes

| **Target** | **Primers** | | **Primer sequence (5'---- 3')** | **Product size (bp)** | **Source** |
| --- | --- | --- | --- | --- | --- |
| ***S. pneumoniae***  **LytA gene** | S. pneu^a^-F^g^: | | TTA TTC GTG CAA TAC TCG TGC G | 319bp | Nagai et al 2001 |
|  | S. pneu^a^-R^h^: | | CAA CCG TAC AGA ATG AAG CGG |  |  |
| ***H. influenzae***  **Hpd gene** | H. inf^b^-F^g^: | | ACT TTA GCC CTT TCT TTA TTA GCA | 1072bp | Wang et al 2011 |
|  | H. inf^b^-R^h^: | | CTT TTA AGA ATT CCA CGC CAG TAT |  |  |
| ***N. meningitidis***  **CtrA gene** | N. men^c^-F^g^: | | ATG CGG TGG CTG CGG TAG GT | 533bp | Guiver et al 2000 |
|  | N. men^c^-R^h^: | | CCG GCG AGA ACA CAA ACG ACA A |  |  |
| **Herpes simplex virus**  **UL30 gene** | HSV^d^-F^g^ | | AGT ACA TCG GCG TCA TCT GC | 403bp | This work* |
|  | HSV^d^-R^h^ | | TAC GGG ATC CGG TCC TTG AT |  |  |
| **Enterovirus**  **5' UTR region** | Ev^e^-F^g^ | CCA TGG GAC GCT TCA ATA CTG ACA | | 250bp | This work* |
|  | Ev^e^-R^h^ | | GGA TGG CCA ATC CAA TAG CTA |  |  |
| **Mumps**  **Fusion protein gene** | MuV^f^-F^g^ | | ACA ATG AGG CAG AGA GGC TG | 250bp | This work* |
|  | MuV^f^-R^h^ | | AAT CCG TCT AGG GAC ACC GT |  |  |

The Primer-BLAST tool was used for primer design. The chosen primer sets were synthesized at the Molecular and Cell Biology Department, University of Cape Town.

^a^ *Streptococcus pneumoniae,* ^b^ *Haemophilus influenzae,* ^c^ *Neisseria meningitidis*, ^d^herpes simplex virus, ^e^enterovirus, ^f^ mumps virus, ^g^ Forward primer, ^h^Reverse primer

Appendix C: PCR assay conditions for amplification of the cloning inserts for the target genes

| **Target** | **Primers and reaction concentration** | **cDNA synthesis and initial denaturation** | **PCR Cycle** | **Final extension** |
| --- | --- | --- | --- | --- |
| *S. pneumoniae*  LytA gene | S. pneu^a^-F^g^: 400nM | 95^o^C (5mins) | 95^o^C (30s)52^o^C(30s) 72^o^C (30s) (X35) | 72^o^C (5mins ) |
|  | S. pneu^a^-R^h^: 400nM |  |  |  |
| *H. influenzae*  Hpd gene | H. inf^b^-F^g^: 400nM | 95^o^C (2mins) | 95^o^C (30s) 55^o^C(30s)72^o^C (90s) (X35) | 72^o^C (5mins ) |
|  | H. inf^b^-R^h^: 400nM |  |  |  |
| *N. meningitidis*  CtrA gene | N. men^c^-F^g^: 400nM | 95^o^C (2mins) | 95^o^C (30s) 56^o^C(30s)72^o^C (30s) (X35) | 72^o^C (5mins ) |
|  | N. men^c^-R^h^: 400nM |  |  |  |
| Herpes simplex virus  UL30 gene | HSV^d^-F^g^ 200nM | 95^o^C (2mins) | 95^o^C (20s) 56^o^C (30) 72^o^C (60s) (X35) | 72^o^C (5mins ) |
|  | HSV^d^-R^h^ 200nM |  |  |  |
| Enterovirus  5' UTR region | Ev^e^-F^g^ 400nM | 42^o^C(15mins) 95^o^C(2mins) | 95^o^C (20s) 52^o^C(30s)72^o^C (60s) (X40) | 72^o^C (5mins ) |
|  | Ev^e^-R^h^ 400nM |  |  |  |
| Mumps  Fusion protein gene | MuV^f^-F^g^400nM | 45^o^C (30s) 95^o^C (1min) | 95^o^C (10s) 56^o^C(30s)72^o^C (30s) (X40) | 72^o^C (5mins ) |
|  | MuV^f^-R^h^ 400nM |  |  |  |

^a^ *Streptococcus pneumoniae,* ^b^ *Haemophilus influenzae,* ^c^ *Neisseria meningitidis*, ^d^herpes simplex virus, ^e^enterovirus, ^f^ mumps virus, ^g^ Forward primer, ^h^Reverse primer

The Go Taq master mix (Promega, United States of America) was used for amplification of the DNA targets and the MyTaq One-step RT-PCR kit (Bioline, London, United Kingdom) was used for the viral targets with cycling conditions as per manufacturer’s recommendations. Gel electrophoresis was carried out on a 1% (w/v) agarose gel at 80 volts for 1hour 30 minutes. The resolved bands were cut out and purified with a MiniElute Gel extraction kit (Qiagen, Hilden, Germany) prior to incorporation into plasmids.

Plasmids were prepared from the purified DNA using the pGEM®-T Easy vector kit and JM109 *E. coli* cells (Promega, United States of America) according to the manufacturer’s protocol. The copy-number of the plasmids was calculated according to Perini and colleugues^149^
